# PFK-158 enhances colistin efficacy against resistant *Edwardsiella piscicida* through synergistic mechanisms

**DOI:** 10.3389/fvets.2026.1748700

**Published:** 2026-02-12

**Authors:** Yajing Pan, Yuepeng Zhang, Zubair Ahmed Laghari, Yangbin Shi, Hans-Peter Grossart, Yelin Jiang, Sihong Wu, Danli Xie, Wanchun Guan, He Zhang, Yongliang Lou, Jinfang Lu

**Affiliations:** 1 Wenzhou Key Laboratory of Sanitary Microbiology, Key Laboratory of Laboratory Medicine, Ministry of Education, China, School of Laboratory Medicine and Life Sciences, Wenzhou Medical University, Wenzhou, Zhejiang, China; 2 Cangnan Ecological Environmental Monitoring Station, Wenzhou Bureau of Ecology and Environment Cangnan Branch, Wenzhou, Zhejiang, China; 3 Department of Veterinary Parasitology, Sindh Agriculture University, Tandojam, Sindh, Pakistan; 4 Leibniz-Institute of Freshwater Ecology and Inland Fisheries (IGB), Stechlin, Germany; 5 Institute of Biochemistry and Biology, University of Potsdam, Potsdam, Germany; 6 Zhejiang Provincial Key Laboratory for Subtropical Water Environment and Marine Biological Resources Protection, National and Local Joint Engineering Research Center of Ecological Treatment Technology for Urban Water Pollution, College of Life and Environmental Sciences, Wenzhou University, Wenzhou, Zhejiang, China

**Keywords:** aquaculture, *Edwardsiella piscicida*, non-antibiotic adjuvant, PFK-158, synergistic effect

## Abstract

*Edwardsiella piscicida* (*E. piscicida*) is a well-known bacterial pathogen that causes severe diseases in various cultured fish species, posing a significant threat to the global aquaculture industry. The increasing incidence of multidrug resistant (MDR) *E. piscicida* has greatly limited the efficacy of conventional antibiotics, highlighting the urgent need for new and effective therapeutic strategies. In the current study, several potential non-antibiotic adjuvants were screened, and PFK-158 was identified as promising compound that synergistically decreased the minimum inhibitory concentration (MIC) of colistin resistant *E. piscicida*, and significantly enhanced colistin's bactericidal activity against *E. piscicida* and other fish pathogens (e.g., *Vibrio parahaemolyticus*). Mechanistic characterization revealed that the combined treatment with PFK-158 and colistin increased bacterial membrane permeability, inhibited efflux pump activity and biofilm formation, promoted colistin accumulation, and induced the overproduction of reactive oxygen species (ROS). Transcriptomic analysis further demonstrated that PFK-158 in combination with colistin significantly downregulated genes associated with bacterial secretion systems, virulence, and lipopolysaccharide modification. Moreover, the co-administration of colistin and PFK-158 efficiently reduced bacterial loads *in vivo* and improved survival rates in infected fish. These results indicate that PFK-158 may serve as a safe and effective colistin adjuvant to synergistically combat MDR *E. piscicida* infections. This study provides valuable insights for the development of non-antibiotic adjuvants to manage diseases caused by *Edwardsiella* and other colistin-resistant pathogens in aquaculture.

## Highlights

- PFK-158 sensitizes *E. piscicida* to colistin, and significantly reduces the MIC;
- The combination of PFK-158 and colistin increases bacterial membrane permeability and induces oxidative stress;
- The combination of PFK-158 and colistin suppress the expression of genes associated with LPS modification, biofilm formation and bacterial virulence;
- Combined therapy of PFK-158 and colistin effectively protects zebrafish from *E. piscicida* infection.

## 1 Introduction

The Gram-negative bacterium *E. piscicida* (formerly designated as *Edwardsiella tarda*) is a well-known intracellular pathogen with a broad host range (1, 2). It is ubiquitously distributed in aquatic habitats and causes Edwardsiellosis, an infectious disease affecting various cultured fish species (e.g., turbot, flounder, and tilapia) as well as other animals hosts (2, 3), and incurs substantial annual economic losses to the global aquaculture industry. Despite the high lethality of edwardsiellosis in infected fish, effective therapeutic strategies are currently limited, antimicrobial agents still serve as the primary intervention for controlling *E. piscicida* infections in aquatic settings (2, 3).

The increasing use of antibiotics in aquaculture has become a major concern, posing a serious threat not only to the mitigation of multidrug-resistant (MDR) but also to global public health. It is estimated that approximately 10,259 tons of antibiotics (e.g., fluoroquinolones, β-lactams, and oxytetracycline) were used in aquaculture solely to control pathogenic infections in 2017, and the amount is expected to rise to 236,757 tons by 2030 owing to the rapid global expansion of the aquaculture industry (4, 5). The misuse and overuse of antibiotics further accelerate the emergence and spread of antibiotic resistance genes (ARGs) and MDR pathogens in aquaculture (6–8), thereby exacerbating the global antibiotic resistance crisis. Specifically, *E. piscicida* isolates from northern Thailand have shown resistance to more than seven antibiotics (9). Moreover, isolates TX01 (10), EIB202 (11), and MS-18-199 (12) harbor transferable MDR plasmids. Consequently, the development of novel and effective strategies to combat bacterial resistance, and mitigate the escalating antibiotic resistance crisis is urgently needed.

The discovery of novel antibiotics is a time-consuming, cost-intensive endeavor characterized by low success rates. Recently, remarkable progress in the development of novel antibiotic adjuvants has been achieved (13, 14). A variety of active compounds have been identified that synergistically enhance the antibacterial activity and therapeutic efficiency of existing antibiotics (15, 16), despite possessing little to no intrinsic bactericidal activity on their own. For instance, auranofin, a U.S. Food and Drug Administration (FDA)-approved drug originally indicated for rheumatoid arthritis, has been repurposed as a broad-spectrum adjuvant capable of resensitizing MDR bacteria to antibiotics (e.g., colistin, carbapenem) (17, 18). Similarly, silver- and bismuth-based compounds (19, 20), as well as antitumor drugs (e.g., daunorubicin) (21–23) have exhibited analogous efficacy, which translates to marked reductions in mortality in infected animal models. Thus, the combinatorial use of conventional antibiotics with such repurposed agents may represent a promising alternative strategy to address the escalating global antimicrobial resistance crisis.

However, most previous studies focused on therapies targeting only a limited number of MDR pathogen species in clinical settings (e.g., *Pseudomonas aeruginosa* and *Klebsiella pneumoniae*) (15, 16). By contrast, few adjuvants have been systematically screened for their effectiveness against MDR pathogens derived from aquatic sources (24–26). In this study, we conducted a preliminary screening of several candidate adjuvants to reverse the intrinsic colistin resistance in *E. piscicida* and found that PFK-158 and carbonyl cyanide 3-chlorophenylhydrazone (CCCP, a well-known protonophore and efflux pump inhibitor) acted synergistically with colistin. The remaining compounds showed limited or no effectiveness. Although CCCP is a proton uncoupler with various side effects and holds limited therapeutic potential (27), PFK-158 has completed a phase I clinical trial demonstrating minimal toxicity (23, 28), and was therefore selected as the most promising adjuvant for further analyses. This study aimed to elucidate the underlying mechanisms governing the synergistic interaction between PFK-158 and colistin and to develop a novel alternative strategy for safeguarding aquatic animals against *E. piscicida* infections.

## 2 Materials and methods

### 2.1 Bacterial strains and reagents

*E. piscicida* PPD130/91 and *Edwardsiella ictaluri* (*E. ictaluri*) were kindly provided by Dr. Haixia Xie (Institute of Hydrobiology, Chinese Academy of Sciences, Wuhan, Hubei, China). *E. piscicida* LY-2019 and ZX-1 were isolated from naturally infected fish. Mutant strains Δ*arnT* and Δ*ugd* were generated from the parental strain PPD130/91 following the method described previously (25). All aforementioned bacterial strains were preserved in tryptic soy broth (TSB) supplemented with 20% glycerol at −80 °C in our laboratory. *In vivo* infection assays were performed using the PPD130/91 strain of *E. piscicida*. Detailed information on the bacterial strains and reagents used in this study is listed in Supplementary Text S1 and Supplementary Table S2, respectively.

### 2.2 Antimicrobial resistance assay

The antibiotic colistin sulfate (colistin) was obtained from MedChem Express (Shanghai, China). All solvents and diluents used in this study were prepared in accordance with the Clinical and Laboratory Standards Institute (CLSI) 2022 guidelines. *E. piscicida* isolates were grown for 12 h in liquid TSB at 28 °C. The MIC of each tested antibiotic was determined using the broth microdilution method as previously described (29). Briefly, overnight bacterial cultures were adjusted to a final concentration of approximately 1 × 106 colony-forming units per milliliter (CFU/ml) and inoculated into 96-well microplates containing twofold serial dilutions of colistin. The plates were then incubated for 18–20 h at 28 °C, and the optical density at 540 nm (OD_540_) was measured using a microplate spectrophotometer (Thermo Fisher Scientific, Waltham, MA, USA). The MIC value was defined as the minimal concentration that completely inhibited visible bacterial growth. All the experiments were performed at least in triplicate.

### 2.3 Checkerboard assay

The synergistic antibacterial activity of the combination of colistin and PFK-158 was evaluated using the checkerboard assay. Different concentrations of colistin and PFK-158 were prepared with a two-fold serial dilution method (8 × 12 matrix). Bacterial strains were routinely propagated in tryptic soy broth (TSB) medium prior to experimental use. For the assay, bacterial culture was inoculated into a sterile 96-well plate at a final density of 1 × 10^6^ CFU/ml and incubated for 18–20 h at 28 °C in three different types of media: CAMHB, host-mimicking Dulbecco's Modified Eagle Medium (DMEM) and LPM [5 mM KCl, 7.5 mM (NH_4_)_2_SO_4_, 0.5 mM K_2_SO_4_, 80 mM MES (pH 5.8), 0.1% casamino acids, 0.3% (v/v) glycerol, 24 μM MgCl_2_, and 337 μM ${\text{PO}}_{4}^{3-}$]. Optical density at 540 nm was recorded for each culture as previously described (30). The antibacterial effects of colistin and PFK-158 were quantified as follows using the fractional inhibitory concentration index (FICi), as described earlier (30):


$$\begin{array}{l} \text{FI}{\text{C}}_{\text{i}}\;=\text{ MI}{\text{C}}_{\text{ab}}/\text{MI}{\text{C}}_{\text{a}}\;+\text{ MIC}{\text{b}}_{\text{ab}}/\text{MI}{\text{C}}_{\text{b}} \end{array}$$

In this study, MIC_a_ represents the MIC of colistin alone; MIC_ab_ represents the MIC of colistin in combination with PFK-158; MIC_b_ represents the MIC of PFK-158 alone; MIC_ba_ represents the MIC of PFK-158 in combination with colistin. A FICi value of ≤ 0.5 indicates a synergistic effect, whereas 0.5 < FICi ≤ 1 denotes a partial synergistic effect, and a FICi value > 1 is considered as antagonistic interaction. Each experiment was performed at least in triplicate.

### 2.4 Time-kill assay

The time-kill assay was conducted to evaluate the synergistic antibacterial activity of colistin in combination with PFK-158. Briefly, an overnight bacterial culture was inoculated into fresh TSB (0.1 %, v/v) and incubated for 24 h at 28 °C. The resulting bacterial suspension was treated with colistin (final concentration of 8 or 16 μg/ml), or PFK-158 (4 μg/ml) alone, or with the combined colistin/PFK-158 treatment for 24 h at 28 °C. During incubation, 100 μl aliquots were collected from each tube at 0, 1, 3, 5, 7, 9, 12, and 24 h. Ten microliter aliquots of each serially diluted sample were plated onto TSB agar plates for colony counting, and the viable bacterial count (CFU/ml) was determined. Synergism between colistin and PFK-158 was considered as a ≥ 2 log_10_ reduction in CFU/ml compared with the most effective mono-treatment. Each experiment was performed at least in triplicate.

### 2.5 Membrane permeability

Outer membrane permeability was assayed using the 1-N-phenylnaphthylamine (NPN) dye following the manufacturer's instructions and previously described protocols (25, 30). Briefly, overnight bacterial cultures were washed and resuspended in 5 mM HEPES (pH 7.2) containing 10 μM NPN, adjusted to an OD_540_ of 0.5 and then treated with PFK-158 (0–8 μg/ml) or colistin (16 μg/ml) at 28 °C. After 4 h of incubation in the dark, the fluorescence intensity of NPN was measured (excitation at 355 nm/emission at 420 nm) using a Synergy NEO2 multifunctional microplate reader (BioTek, Vermont, USA). Each experiment was performed in triplicate.

Inner membrane integrity was evaluated by staining with propidium iodide (PI; 20 μM) following a previously published method (25). Bacterial cells were cultured and treated as described above, and the fluorescence intensity of PI-labeled cells was measured (excitation at 488 nm/emission at 615 nm) using a microplate reader. Each experiment was performed at least in triplicate.

### 2.6 Total ROS analysis

2′7′-dichlorodihydrofluorescein diacetate (DCFH-DA) probe was used to detect intracellular ROS levels in *E. piscicida* as previously described (29). Briefly, pre-treated *E. piscicida* suspensions were incubated with a final concentration of 5 μM DCFH-DA in the dark for 2 h with shaking, after which the fluorescence intensity was measured (excitation at 488 nm/emission at 525 nm) using a microplate reader. Each experiment was performed at least in triplicate.

### 2.7 Scanning electron microscope (SEM) and transmission electron microscopy (TEM)

The morphological changes of treated bacterial cells were observed using SEM and TEM according to the methods described (30, 31). Briefly, treated *E. piscicida* PPD130/91 cells were collected after 24 h by centrifugation (6,000 g, 5 min, 4 °C) and fixed for an additional 24 h at 4 °C in 2.5% (v/v) glutaraldehyde solution. The cells were then washed three times with phosphate-buffered saline (PBS, 0.1 M), and fixed for another 2 h in osmic acid solution. Subsequently, the fixed *E. piscicida* cells were dehydrated through an ethanol series (30%−100%). Finally, a portion of the treated cells was sputtered with gold for SEM observation. The remaining dehydrated cells were embedded, and ultrathin sectioned using an ultramicrotome and scanned by a TEM after staining with 2% uranyl acetate and Reynolds' lead citrate.

### 2.8 H33342 accumulation assay

Accumulation assays using Hoechst 33342 (H33342) were performed to evaluate the inhibitory effect of PFK-158 on bacterial efflux pump activity following a previous study (32) with slight modifications. First, the cytotoxicity of H33342 was assessed to determine a non-toxic working concentration. *E. piscicida* at the mid-logarithmic growth phase was incubated with 2.5, 10, and 50 μM H33342, and samples were collected at 0, 30, 60, and 120 min for colony counting on TSB agar plates. The preliminary results indicated that 2.5 μM H33342 was an appropriate concentration for subsequent experiments. Hence, *E. piscicida* at the logarithmic growth phase (OD_540_ ≈ 0.5) were adjusted to OD_540_ ≈ 0.1, and treated with PFK-158 at a concentration ranging from 2 to 8 μg/ml. Aliquots (180 μl each) were transferred to a 96-well black micro-plate and H33342 was added to achieve a final concentration of 2.5 μM. The fluorescence intensity of H33342 was detected (excitation at 355 nm/emission at 460 nm) using a microplate reader for 30 cycles with 75 s intervals between readings.

### 2.9 Colistin accumulation assay

The intracellular concentration of colistin was measured using an enzyme-linked immunosorbent assay (ELISA) kit (Abebio, Wuhan, China) following the manufacturer's instructions. Briefly, *E. piscicida* in mid-logarithmic growth phase was treated with colistin alone (8 or 16 μg/ml) or in combination with PFK-158 (4 μg/ml) at 28 °C for 6 h. Bacterial cells were collected by centrifugation (6,000 × g, 5 min, 4 °C) and washed three times with 0.9% (w/v) sterile normal saline (NaCl). The pellets were resuspended in normal saline and adjusted to an OD540 of 1.0 to normalize bacterial cell densities across all experimental samples. Finally, bacterial cells were disrupted by ultrasonication on ice (200 W, 5 s on/5 s off, 3 min total), and the supernatants obtained after centrifugation (10,000 × g, 5 min, 4 °C) were used for quantification of intracellular colistin. Each experiment was performed at least in triplicate.

### 2.10 Transcriptome analysis

*E. piscicida* PPD130/91 cells in the mid-logarithmic growth phase (1 × 10^7^ CFU/ml) cultured in CAMHB medium were treated with colistin alone or in combination with PFK-158 for 6 h, with the colistin only treatment serving as the control group. The bacterial cells were collected by centrifugation (6, 000 × g, 5 min, 4 °C) and washed three times with PBS. Finally, three independent replicates were prepared, and bacterial pellets were immediately frozen in liquid nitrogen and promptly shipped to Majorbio Biotechnology Co., Ltd. (Shanghai, China) for transcriptome sequencing and bioinformatic analysis. Genes exhibiting an absolute fold change (|FC|) ≥ 2 and a Bonferroni-corrected *p* value (*p*_adj_) of < 0.05 were defined as differentially expressed genes (DEGs). Quantitative real-time polymerase chain reaction (qRT-PCR) analysis was used to validate the expression patterns of selected DEGs. *16S rRNA* was used as the reference gene, and the relative expression levels were calculated using the 2^−ΔΔ*CT*^ method (33). Additional details regarding the transcriptomic and qRT-PCR analysis are provided in Supplementary Text S3 and Supplementary Table S3.

### 2.11 Zebrafish infection model

Healthy adult zebrafish (*Danio reri*o) with a standard length of 2.5–3.0 cm and body weight of 0.20–0.25 g were randomly allocated into five groups (26–27 fish per group). For infection, zebrafish in all treatment groups were intraperitoneally (i.p.) injected with 10 μl of *E. piscicida* PPD130/91 suspension at a dose of 2 × 105 CFU per fish, according to previous studies (25, 32). Fish in the negative control group (1) received an i.p. injection of 10 μl of normal saline only, without bacterial challenge. One hour after bacterial infection, fish were intraperitoneally administered with (2) normal saline (0.9%, NaCl; vehicle group), (3) colistin (8 mg/kg body weight), (4) PFK-158 (10 mg/kg body weight), or (5) colistin combined with PFK-158 (8 mg/kg colistin + 10 mg/kg PFK-158). After 24 h post infection, a subset of zebrafish (*n* = 6 per group) were euthanized with a rapid cooling method (2–4 °C, ice–water bath) as previously described (32). Tissue samples of the liver, spleen, kidney, intestine, and gills were immediately dissected from euthanized fish for subsequent bacterial load quantification. The survival status of the remaining zebrafish in each group was monitored daily for 7 consecutive days post infection. Zebrafish were resumed on feeding at 48 h post-infection to maintain normal physiological conditions during the survival assay.

### 2.12 Statistical analysis

Statistical analysis was performed using GraphPad Prism 9.0 and SPSS software. All results are presented as mean ± standard deviation (SD). For data following a normal distribution (verified using the Shapiro–Wilk test), one-way or two-way analysis of variance (ANOVA) was conducted. For non-normally distributed data, the Mann–Whitney *U* test was applied. Differences in zebrafish survival were evaluated using the log-rank (Mantel-Cox) test. Statistical significance was defined as *p* < 0.05, with significance levels represented as ^*^p < 0.05, ^**^p < 0.01, and ^***^p < 0.001.

## 3 Results

### 3.1 Primary screening of candidate adjuvants with colistin *in vitro*

**Figure 1:**
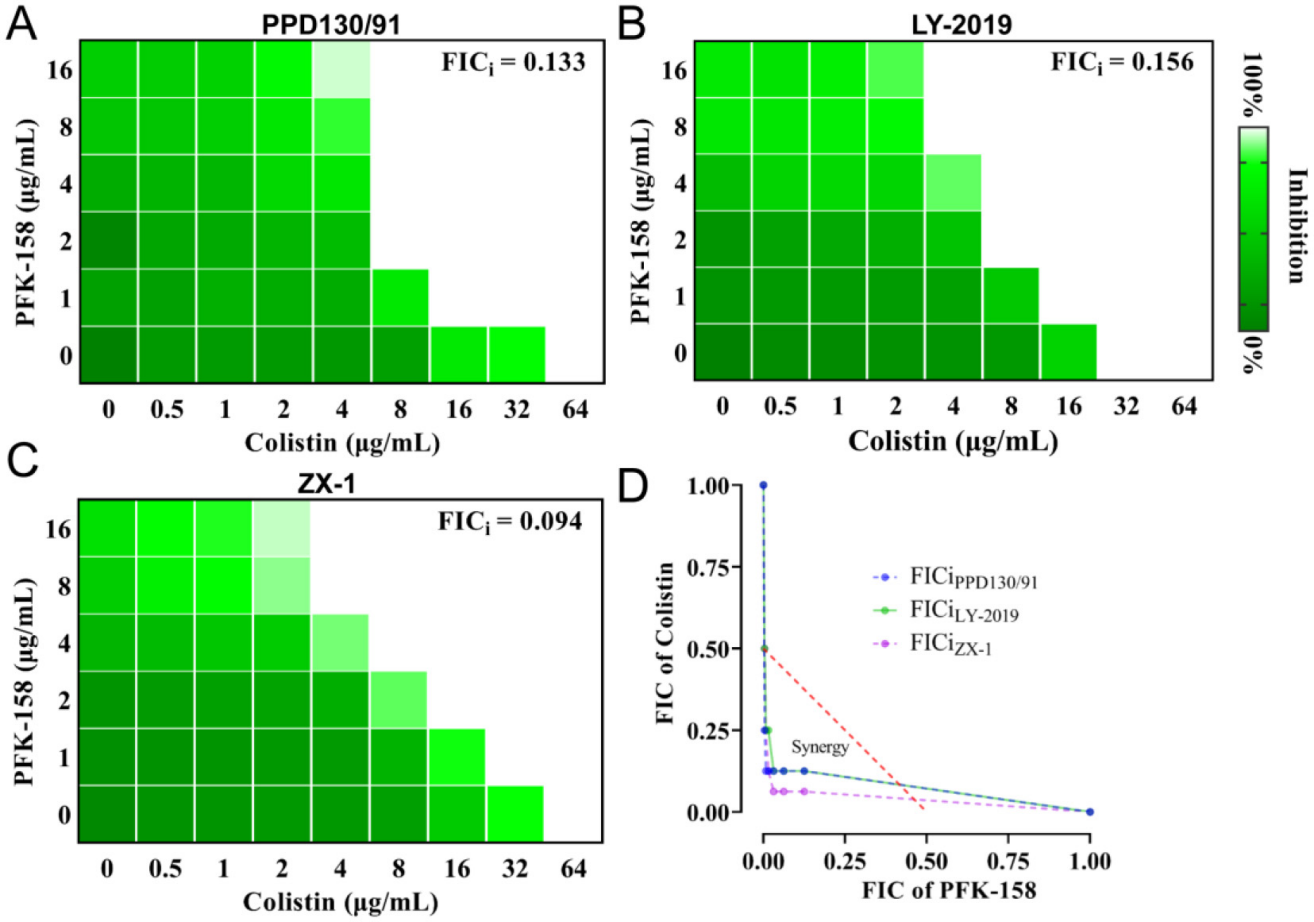
PFK-158 in combination with colistin significantly restores the colistin susceptibility in colistin-resistant *E. piscicida*. **(A–C)** Heatmaps generated from micro-dilution checkerboard assays illustrating the interaction between PFK-158 and colistin against three *E. piscicida* isolates (PPD130/91, LY-2019, ZX-1). Dark green regions represent higher bacterial cell density, corresponding to weaker antibacterial activity. **(D)** Isobolograms depicting the synergistic interaction of PFK-158 and colistin against three different *E. piscicida* isolates. The red dashed line indicates an ideal isobole, corresponding to additive or independent drug action. Data points below this line (FICi < 0.5) indicate a synergistic effect between the two compounds.

Primary screening of six candidate adjuvants showed MICs of each *E. piscicida* strain against these compounds ranged from 2 to 256 μg/ml. All isolates exhibited high sensitivity to carbonyl cyanide m-chlorophenylhydrazone (CCCP) but displayed markedly greater resistance to PFK-158 and the remaining candidate compounds (Table 1 and Supplementary Table S1). PFK-158 alone exhibited no detectable antimicrobial activity against any of the tested isolates (MIC values were all ≥ 256 μg/ml; Table 1 and Supplementary Table S1). Checkerboard assays indicated that the colistin/PFK-158 combination reduced the MIC_colistin_ of *E. piscicida* PPD130/91 from 64 to 8 μg/ml (an eight-fold reduction; Table 2). Likewise, this combination also decreased the MIC_colistin_ of strains LY-2019 and ZX-1 from 32 to 8 μg/ml and from 64 to 4 μg/ml, respectively (Table 2). The corresponding FICi values for strains PPD130/91, LY-2019, and ZX-1 were 0.133 (synergistic), 0.156 (synergistic), and 0.094 (synergistic), respectively (Figure 1; Table 2). The colistin/CCCP combination also exhibited significant synergistic activity against *E. piscicida* (Table 2). In contrast, the remaining candidate adjuvants, epigallocatechin-3-gallate (EGCG), quercetin, myricetin, and Phe-Arg-β-naphthylamide dihydrochloride (PAβN), failed to exhibit significant synergistic effects when combined with colistin (Table 1 and Supplementary Table S1). Given that CCCP functions as a protonophore with multiple off-target effects and no established therapeutic efficacy, whereas PFK-158 is an antitumor agent that has successfully completed a Phase I clinical trial with minimal observed toxicity, PFK-158 was selected as the most promising adjuvant for subsequent mechanistic and *in vivo* investigations.

**Table 1:** Antimicrobial effect of colistin against *E. piscicida* (PPD130/91) in the presence of different candidate adjuvants.

**Candidate adjuvants**		**Chemical structure**	**MIC^a^ (μg/ml)**	**MIC^b^ (μg/ml)**	**FIC index**	**Potentiation (fold)^c^**
Natural metabolites	EGCG	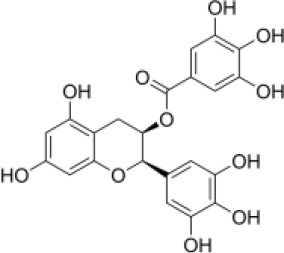	64	64	>1	–
	Quercetin	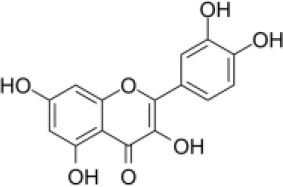	64	64	>1	–
	Myricetin	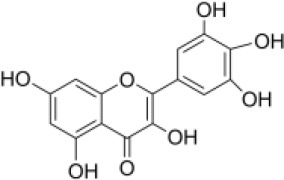	64	256	>1	–
Synthetic compounds	CCCP	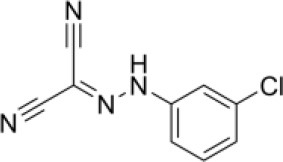	64	0.25	0.313	256
	PAβN	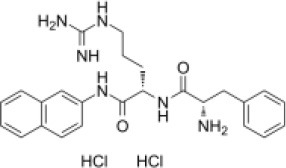	64	64	>1	–
	PFK-158	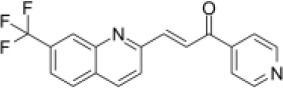	64	8	0.133	8

^a^MICs of COL in the absence of adjuvants.

^b^MICs of COL in the presence of adjuvants.

^c^Potentiation (fold), degree of colistin potentiation in the presence of each adjuvant.

**Table 2:** MIC values of colistin in combination with PFK-158 for each of the three tested bacterial isolates.

**Isolates**	**MIC of mono-therapy (**μ**g/ml)**		**MIC of combination (**μ**g/ml)**		**Potentiation (fold)^a^**	**FIC index^b^**	**Interpretation**
	**Colistin**	**PFK-158**	**Colistin**	**PFK-158**			
PPD130/91	64	>256	8	2	8	0.133	Synergy
LY-2019	32	>256	4	8	8	0.156	Synergy
ZX-1	64	>256	4	8	16	0.094	Synergy

^a^Potentiation (fold), degree of colistin potentiation in the presence of PFK-158.

^b^FIC index, fractional inhibitory concentration index.

### 3.2 PFK-158 restored colistin activity against colistin-resistant bacteria

**Figure 2:**
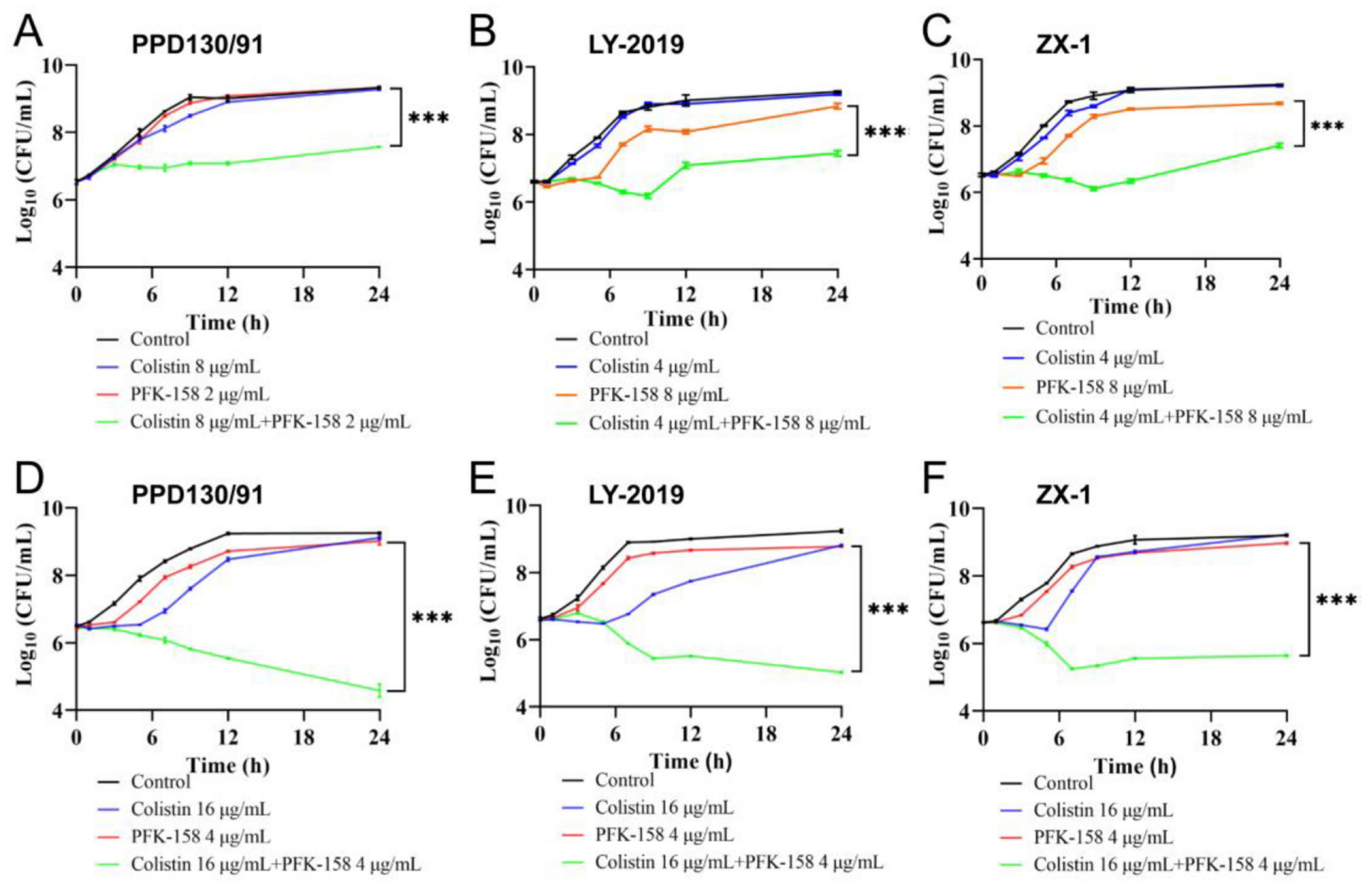
The combination of PFK-158 and colistin exhibits great bactericidal activity against colistin-resistant *E. piscicida*. **(A, D)** Growth curves of strain PPD130/91. **(B, E)** Growth curves of strain LY-2019. **(C, F)** Growth curves of strain ZX-1. *E. piscicida* cells at mid-logarithmic growth phase were treated with colistin or PFK-158 alone, or their combination at the indicated concentrations at 28 °C for 24 h. Colony-Forming Units (CFU) counts were performed at the indicated time points. Results are shown as mean ± standard deviation (SD). Statistical significance was evaluated by two-way ANOVA analysis and shown with ***p < 0.001.

Strains PPD130/91, LY-2019, and ZX-1 exhibited robust growth profiles when treated individually with colistin (2–16 μg/ml) or PFK-158 (2 or 4 μg/ml) in TSB medium (Figure 2). No statistically significant differences in growth were observed among these groups after 24 h (Figure 2). In contrast, the treatment with a combination of colistin (16 μg/ml) and PFK-158 (4 μg/ml) reduced the surviving populations of PPD130/91, LY-2019, and ZX-1 by 4.67, 4.21, and 2.99 log_10_ CFU/ml, respectively, after 24 h (Figures 2D–F). The colistin/PFK-158 combination also exhibited strong synergistic effects against *E. ictaluri, Edwardsiella anguillarum* (*E. anguillarum*), *Vibrio parahaemolyticus, Vibrio vulnificus*, and *Escherichia coli* (*E. coli*) DH5α (MCR-1; Supplementary Figures S1A–G), except for the empty vector containing strain *E. coli* DH5α (pJN105; Supplementary Figure S1H). These results suggest that PFK-158 in combination with colistin effectively reverse colistin resistance phenotypes among various fish pathogens *in vitro*, showing broad-spectrum synergistic antibacterial activity.

Checkerboard assays were further performed using host-mimicking culture media, including DMEM and the LPM medium. The colistin/PFK-158 combination significantly reduced the MIC_colistin_ of *E. piscicida* PPD130/91 from 64 to 16 μg/ml in DMEM medium and from 32 to 2 μg/ml in LPM medium (Supplementary Figure S2). The FICi values were 0.281 and 0.094 (both < 0.5), suggesting an excellent synergistic interaction between colistin and PFK-158 under both conditions. Therefore, this combination shows promising application potential for the treatment of MDR bacterial infections *in vivo*.

### 3.3 The combination of PFK-158 and colistin enhanced the outer membrane permeability

**Figure 3:**
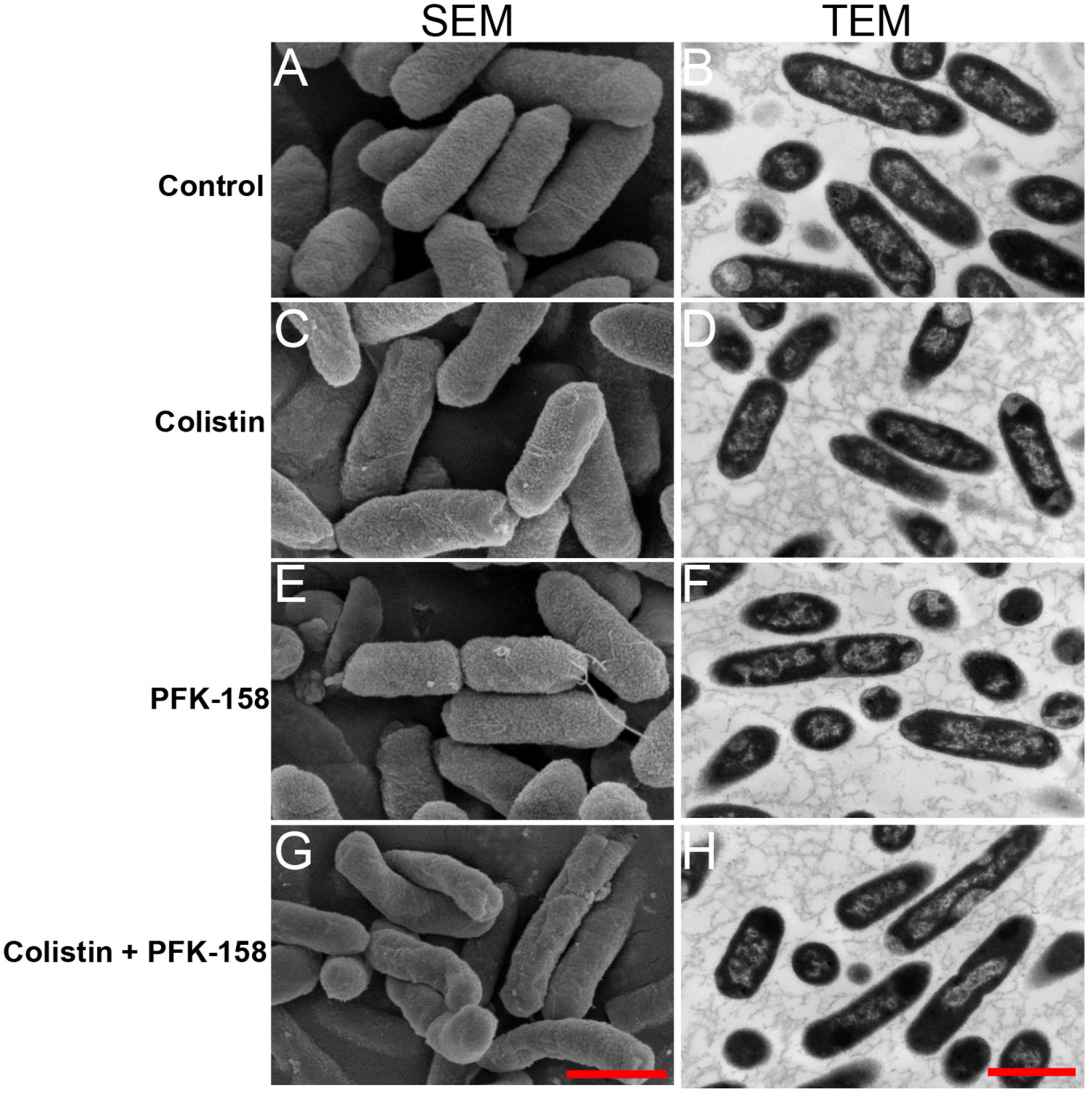
Morphological changes of *E. piscicida* PPD130/91 following different treatment. **(A, C, E, G)** SEM images of *E. piscicida*. Scar bar, 1 μm. **(B, D F, H)** TEM images of *E. piscicida*. Scar bar, 1 μm. **(A, B)** Untreated control group; **(C, D)** Colistin (16 μg/ml) monotherapy group; **(E, F)** PFK-158 (4 μg/ml) monotherapy group; **(G, H)** Colistin (16 μg/ml) combined with PFK-158 (4 μg/ml) group.

**Figure 4:**
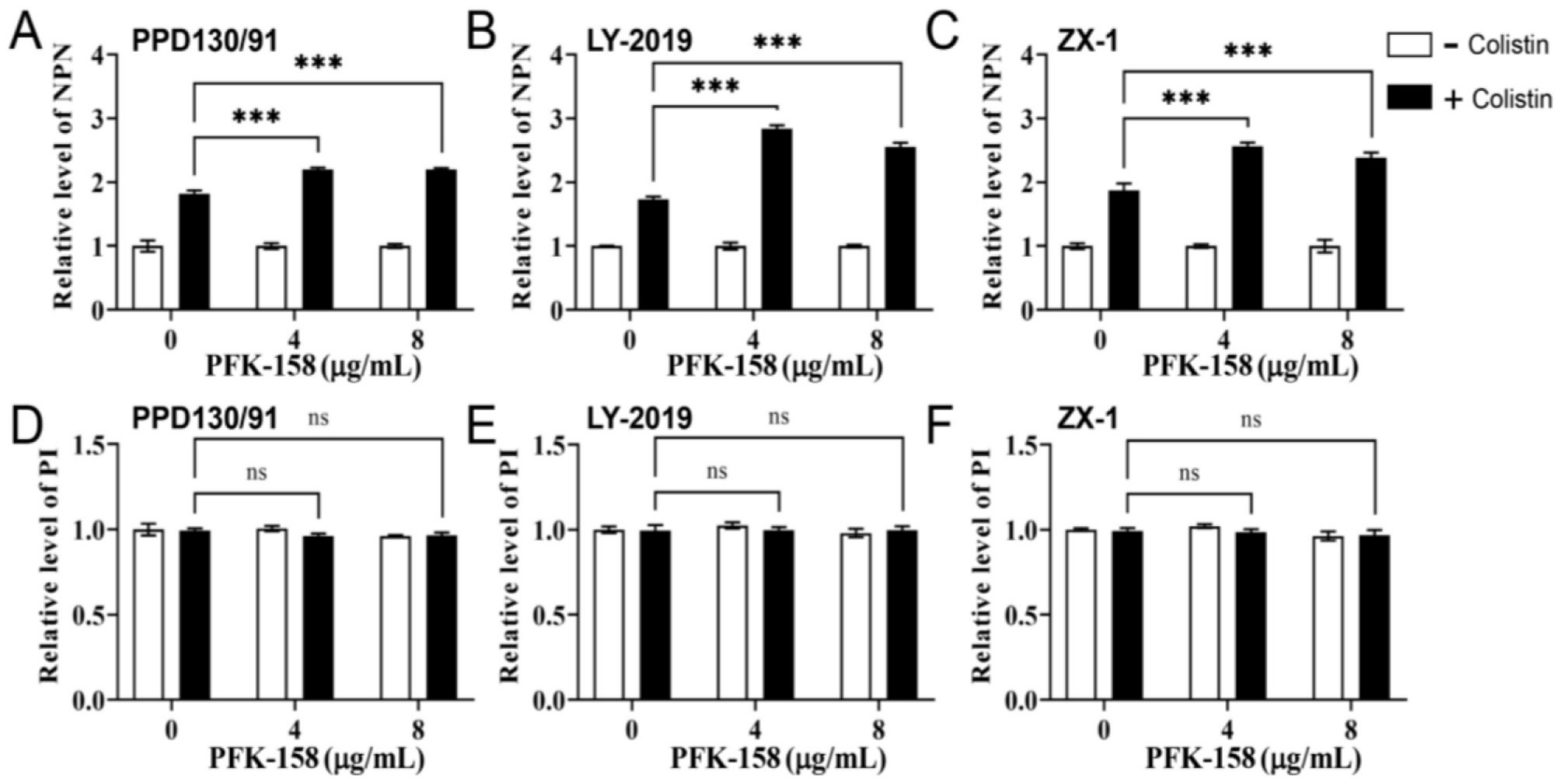
PFK-158 potentiates the membrane-damaging activity of colistin and triggers oxidative stress in *E. piscicida*. **(A–C)** Outer membrane (OM) permeability of colistin-resistant *E. piscicida* isolates following 4 h of treatment with PFK-158 alone, colistin alone, or the colistin-PFK-158 combination. OM permeability was evaluated by measuring the fluorescence intensity of NPN probe, with higher fluorescence indicating increased permeability. **(D–F)** Inner membrane permeability of *E. piscicida* isolates exposed to PFK-158 alone, or PFK-158 combined with colistin for 4 h, respectively. Membrane permeability was evaluated by the fluorescence intensity of the PI probe, where higher fluorescence reflects compromised integrity. Significant differences were evaluated by two-way ANOVA analysis (***p < 0.001, ns, no statistical significance).

The results show that, neither PFK-158 (4 μg/ml) nor colistin (16 μg/ml) administered alone induced any obvious morphological changes in *E. piscicida* PPD130/91 cells compared with the control group (Figures 3A, C, E). In contrast, treatment with the colistin-PFK-158 combination elicited varying degrees of concave deformation and surface shrinkage in the bacterial cells (Figure 3G). Notably, TEM revealed no discernible morphological changes in the *E. piscicida* cell membrane under any of the tested treatment conditions (Figures 3B, D, F, H). Biochemical assays, however, demonstrated that combined treatment with 4 μg/ml PFK-158 and colistin increased outer membrane (OM) permeability by 20.95, 64.56, and 37.05% in strains PPD130/91, ZX-1, and LY-2019, respectively, after 4 h of incubation, compared with colistin monotherapy (Figures 4A–C). When the concentration of PFK-158 was increased to 8 μg/ml, the combination further enhanced OM permeability after 4 h by 20.84, 47.72, and 27.70% in PPD130/91, ZX-1 and LY-2019 (Figures 4A–C), respectively, relative to colistin alone. In stark contrast, the colistin/PFK-158 combination did not significantly enhance the IM permeability (Figures 4D–F) compared with the control or single agent treatments. These data suggest that PFK-158 significantly enhance the OM-damaging activity of colistin against *E. piscicida* isolates, a mechanism that likely contributes to the enhanced bactericidal efficacy of the colistin-PFK-158 combination.

### 3.4 The colistin/PFK-158 combination inhibited efflux pump, promoted accumulation of intracellular colistin, and stimulated ROS

**Figure 5:**
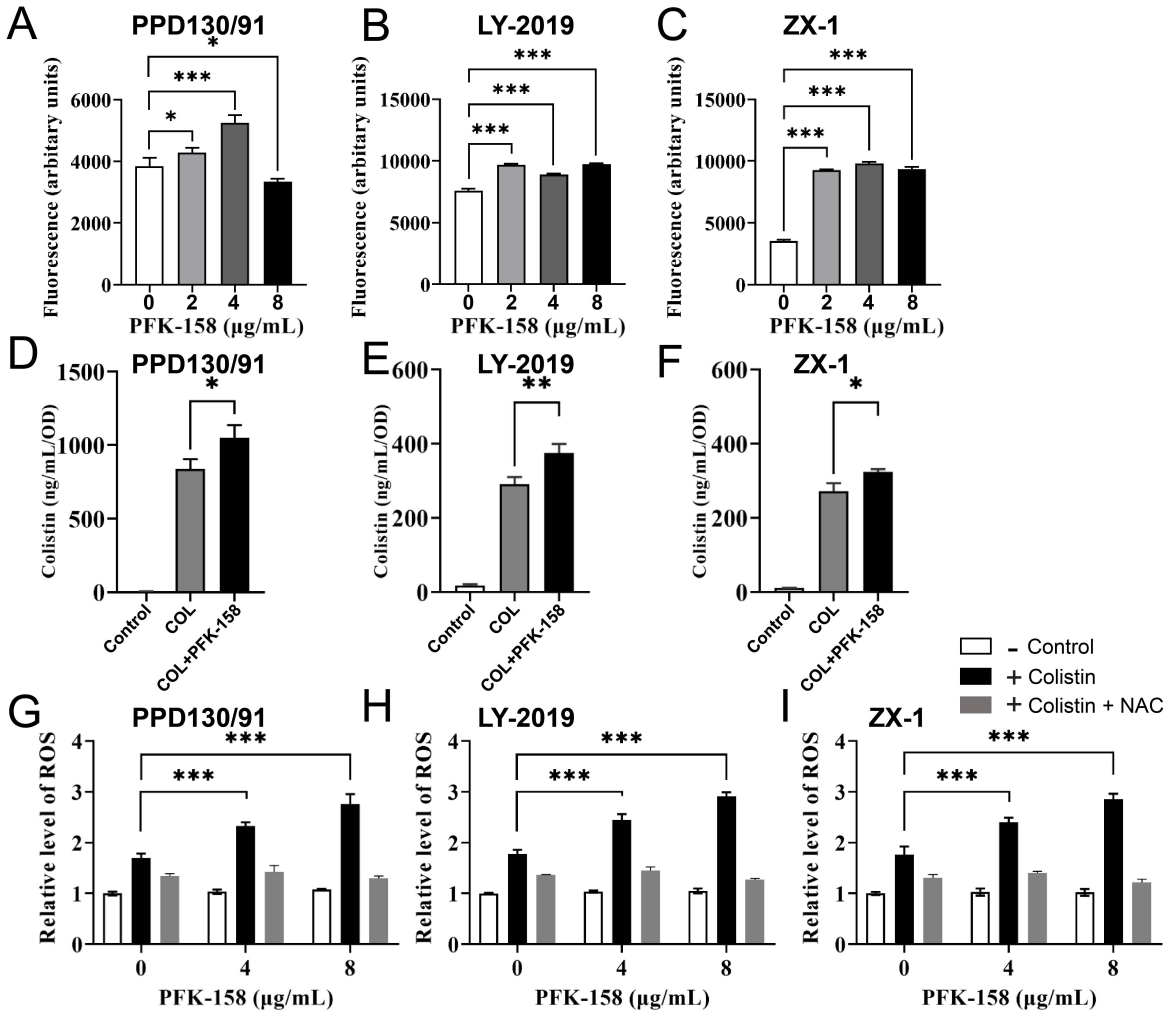
PFK-158 inhibits efflux pump activity, promotes intracellular accumulation, and induces ROS generation in *E. piscicida*. **(A–C)** Inhibitory effect of PFK-158 (2–8 μg/ml) on efflux pump activity in three *E. piscicida* isolates. Efflux activity was evaluated by measuring the fluorescence intensity of the accumulation of the DNA staining probe H33342, where higher fluorescence indicates reduced efflux activity and increased probe accumulation. **(D–F)** Promotion of intracellular colistin accumulation by PFK-158 in the three *E. piscicida* isolates. Intracellular colistin content was assayed by ELISA. **(G–I)** ROS generation in *E. piscicida* isolates following 4 h of treatment with PFK-158 alone (without colistin), the colistin-PFK-158 combination, or the combination plus 5 mM N-acetyl-L-cysteine (NAC, ROS scavenger). Intracellular ROS levels were quantified using the DCFH-DA probe, with higher fluorescence reflecting elevated ROS levels. Significant differences were evaluated by one-way ANOVA analysis (*p < 0.05, **p < 0.01, ***p < 0.001, ns, no statistical significance).

Bacterial efflux activity was evaluated using the H33342 accumulation assay. As shown in Figures 5A–C, significant accumulation of H33342 was observed in all three *E. piscicida* strains following treatment with PFK-158 alone, demonstrating that PFK-158 exerts a potent inhibitory effect on bacterial efflux pump function. Concordantly, combined treatment with the colistin-PFK-158 combination resulted in a statistically significant increase in intracellular colistin concentrations by 24.97, 29.06, and 19.16% in strains PPD130/91, LY-2019, and ZX-1, respectively, relative to colistin monotherapy (Figures 5D–F), compared with the colistin monotherapy.

In parallel, co-treatment with colistin and 4 μg/ml PFK-158 induced marked overproduction of ROS, with ROS levels increasing by 37.12, 37.65, and 35.84% in PPD130/91, LY-2019, and ZX-1 (Figures 5G–I), respectively, relative to colistin alone treatment. Increasing the PFK-158 concentration to 8 μg/ml further augmented ROS levels by 62.88, 63.74, and 61.56% (Figures 5G–I) in the corresponding strains. Notably, supplementation with 5 mM N-acetyl-L-cysteine (NAC) effectively abrogated ROS overproduction, restoring levels to those comparable with the untreated control groups (Figures 5G–I). Collectively, these findings elucidate a sequential mechanistic cascade: PFK-158 inhibits bacterial efflux pump activity, thereby enhancing intracellular colistin accumulation; this, in turn, promotes excessive ROS generation and induces oxidative damage in bacterial cells.

### 3.5 The colistin/PFK-158 combination inhibited the bacterial secretion and virulence systems, two-component system (TCS), flagellar assembly, lipopolysaccharide (LPS) modification and biofilm formation

**Figure 6:**
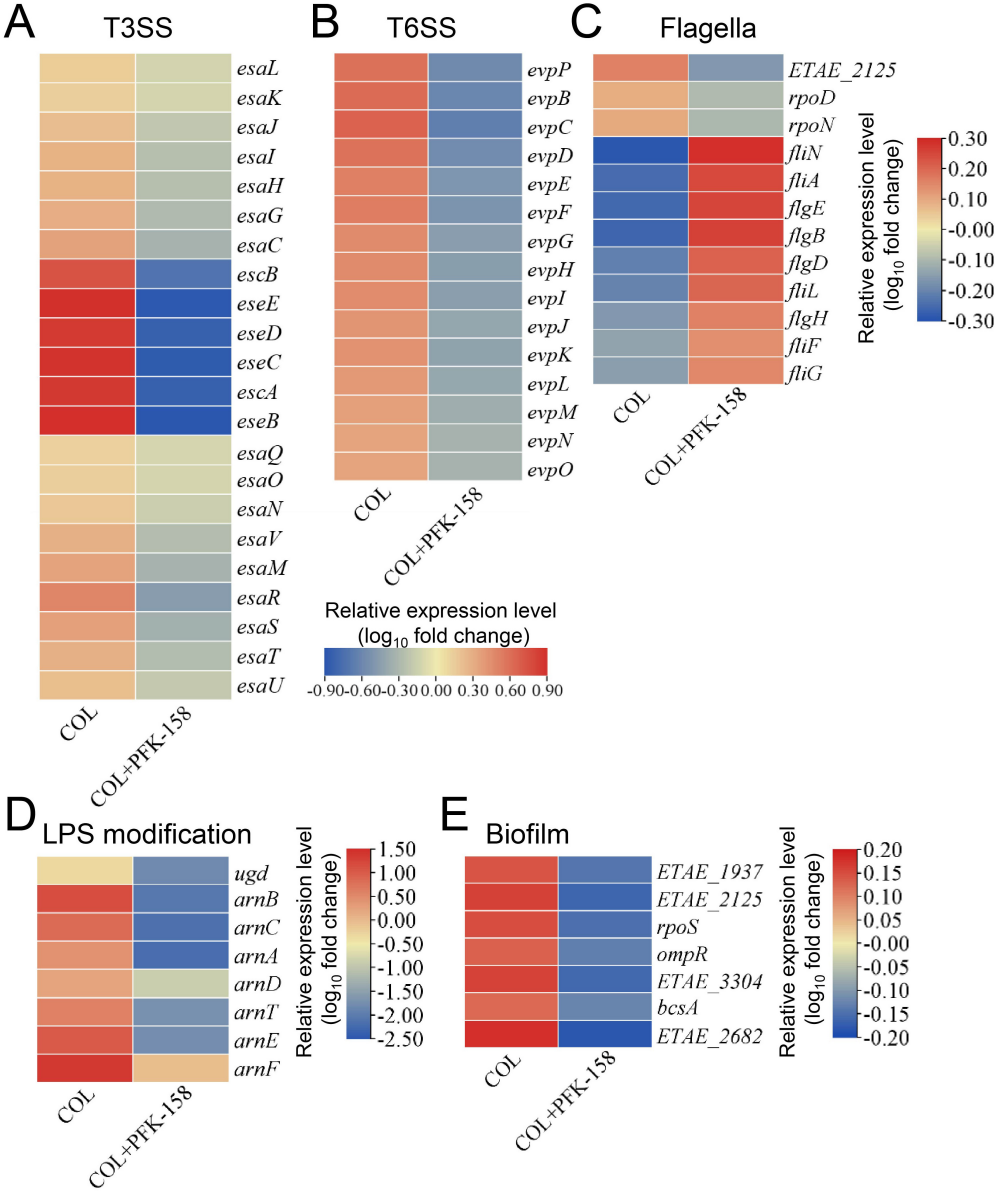
Effect of the combination of PFK-158 with colistin on transcriptional changes in *E. piscicida* genes. **(A–E)** Selected differentially expressed genes involved in T3SS and T6SS, flagellar, LPS modification, and biofilm formation. Red represents upregulated genes; blue indicates downregulated genes. COL, colistin monotherapy; COL+PFK-158, colistin-PFK-158 combination therapy.

Considering that the combination of colistin and PFK-158 exhibited a much stronger antibacterial effect than colistin monotherapy group (serving as the control), we focused on identifying DEGs between the colistin/PFK-158 treated and colistin alone (control) groups. The DEGs were significantly enriched in pathways pertaining to bacterial secretion and virulence systems, flagellar assembly, LPS modification, biofilm formation and two-component system (TCS) pathways (Figure 6 and Supplementary Figure S3). Specifically, genes involved in the bacterial type III secretion system (T3SS; e.g., *esaJ, eseG*, and *eseE*) and type VI secretion system (T6SS; e.g., *evpP, evpC*, and *evpJ*) were remarkably downregulated in the combination treatment group (Figures 6A, B). Furthermore, the combination also significantly suppressed expression of the *arnBCADTEF* operon and *ugd* (encoding UDP-glucose dehydrogenase), which are responsible for LPS biosynthesis and modification (Figure 6D). Additionally, the expression of several biofilm-associated genes (e.g., *rpoS, bcsA, ETAE-1937, ETAE-3304*, and *ETAE-2682*) was simultaneously downregulated (Figure 6E), and biofilm formation was remarkably inhibited by the combination treatment (Supplementary Figure S4). The expression levels of 25 representative DEGs (e.g., *ugd, arnT, evpP*, and *rpoS*) were validated via qRT-PCR (Supplementary Figure S4C), and their expression profiles were consistent with those derived from RNA-sequencing (RNA-seq).

Consistently, PFK-158 significantly enhanced the binding affinity of colistin for the bacterial cell membrane in a concentration-dependent manner (Supplementary Figure S5). For instance, as the PFK-158 concentration increased from 0 to 2, 4, 8, and 16 μg/ml, the membrane-bound colistin level rose concomitantly from 19.97 to 30.35, 43.85, 57.87, and 81.84 ppb (Supplementary Figure S5), respectively. When the colistin concentration was 32 μg/ml, PFK-158 still enhanced the membrane binding of colistin by 1.17-, 1.69-, 2.00-, and 2.38-fold at PFK-158 concentrations of 2, 4, 8, and 16 μg/ml, respectively, compared to the control group (Supplementary Figure S5).

### 3.6 PFK-158 enhanced colistin efficacy against *E. piscicida* infection *in vivo*

**Figure 7:**
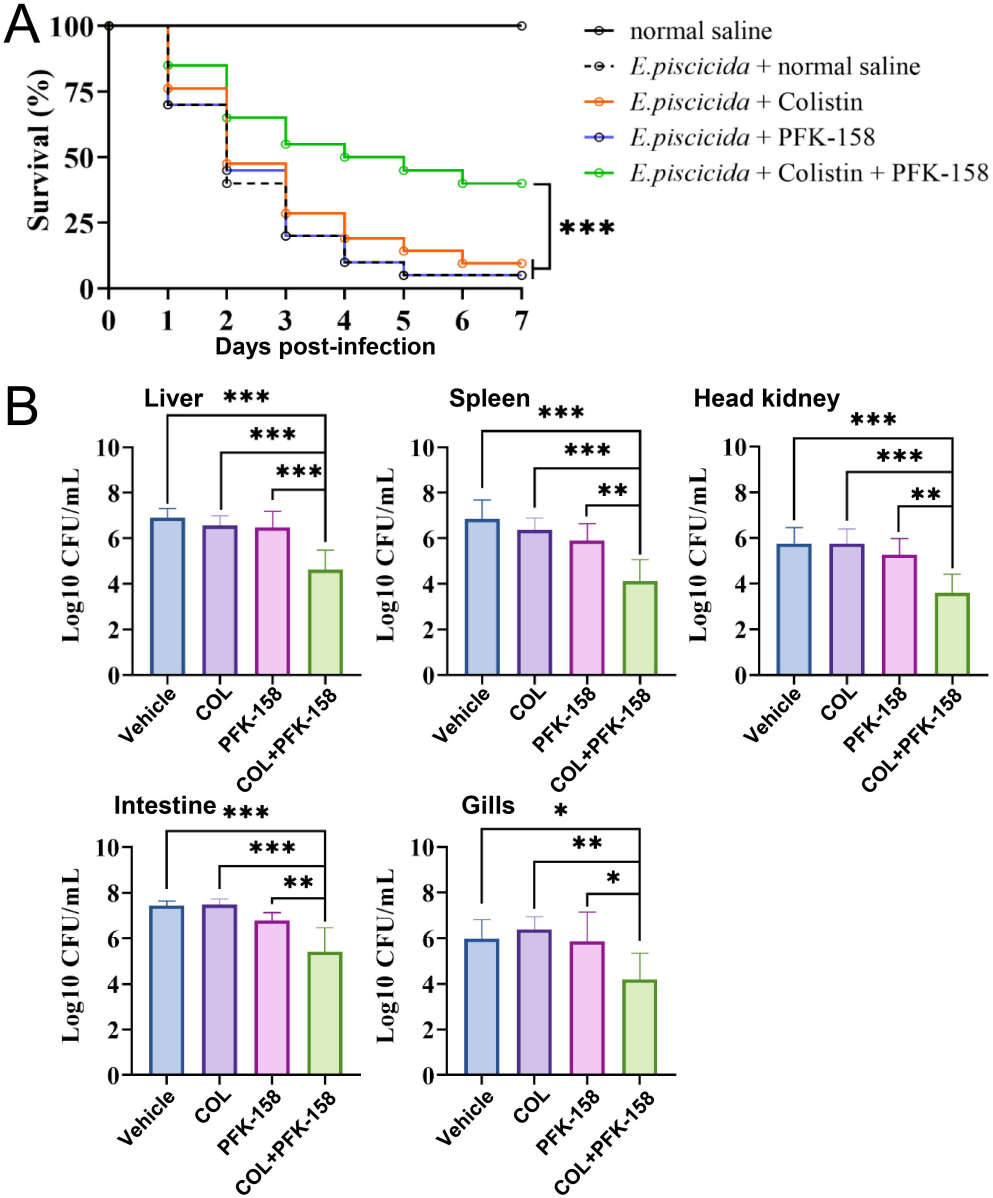
The combination of PFK-158 and colistin enhances the bactericidal activity of colistin against *E. piscicida in vivo*. **(A)** Survival curves of *E. piscicida*-infected zebrafish with different treatments. Adult zebrafish were intraperitoneally (i.p.) infected with *E. piscicida* PPD130/91, then treated with normal saline (vehicle control), colistin (8 mg/kg body weight) alone, PFK-158 (10 mg/kg body weight) alone, colistin combined with PFK-158 (8 + 10 mg/kg body weight), respectively. **(B)** Bacterial loads in the liver, spleen, head kidney, intestine, and gill of infected zebrafish in each treatment group. The significance of zebrafish survival rate and bacterial loads were analyzed by the log-rank (Mantel–Cox) test, and the Mann–Whitney *U* test, respectively (*p < 0.05, **p < 0.01, ***p < 0.001, ns, no statistical significance).

The therapeutic efficacy of the colistin/PFK-158 combination was evaluated using a zebrafish infection model. Infected zebrafish exhibited typical clinical manifestations of edwardsiellosis, including external signs (skin ulceration and hemorrhage) and internal pathologies (abdominal distension and visceral organ damage; Supplementary Figures S6B–D). In stark contrast, combined treatment with colistin (8 mg/kg) and PFK-158 (10 mg/kg) markedly ameliorated these clinical signs (Supplementary Figure S6E) and significantly improved the survival rate of infected zebrafish (Figure 7A). By comparison, monotherapy with either PFK-158 or colistin failed to confer any protective effect against *E. piscicida* infection. Specifically, only one zebrafish survived until 7 days post-infection in both the saline-treated control group (5.0%, 1/20) and the PFK-158 monotherapy group (5.0%, 1/20). Similarly, merely two zebrafish remained alive in the colistin monotherapy group (9.5%, 2/21). In stark contrast, the combination treatment group achieved a markedly higher survival rate of 40.0% (8/20) over the same observation period (Figure 7A). Furthermore, the combined colistin/PFK-158 treatment also significantly decreased the bacterial loads in the liver, spleen, kidney, intestine, and gill of infected zebrafish by 2.29-, 2.73-, 2.16-, 2.02-, and 1.79 –log_10_ CFU/ml, respectively (Figure 7B) compared with the vehicle control. Taken together, these findings indicate that PFK-158 effectively restores the *in vivo* efficacy of colistin against intrinsically colistin-resistant *E. piscicida*, thereby validating the robust therapeutic potential of this combination strategy.

## 4 Discussion

Aquaculture is increasingly acknowledged as a major environmental reservoir for antibiotic-resistant genes (including *mcr* genes) (7, 34), and the emergence of MDR bacteria in clinical settings is closely associated with their presence in aquaculture environments (8, 35, 36). As a result, there is an urgent need to develop alternative strategies to control infections and prevent the spread of aquatic pathogens. In the present study, we investigated how several known adjuvants interact with colistin against *E. piscicida*. Among all the tested compounds, PFK-158 showed a significant synergistic effect (FICi < 0.5) with colistin (Tables 1, 2, Figure 1). In contrast, EGCG, quercetin, myricetin, and PAβN exhibited no apparent synergistic effect against *E. piscicida* (Table 2), even though previous studies have reported their synergistic antibacterial activity along with antibiotics (37–40). This unexpected result highlights the importance of specificity in adjuvants for individual pathogen species. Interestingly, the combination of PFK-158 and colistin exhibited broad synergistic effects against multiple aquatic pathogens (Supplementary Figure S1), thereby expanding their therapeutic spectrum. Unlike previous studies focusing on colistin resistant Gram-negative bacteria of clinical origin (23, 28), our findings extend this synergy to bacteria associated with aquaculture settings.

Moreover, the combination of colistin and PFK-158 demonstrated a significant therapeutic effect in the zebrafish infection model (Figure 7). A single administration of the colistin/PFK-158 combination significantly reduced bacterial loads in important organs and strikingly improved the survival rate of the fish (Figure 7). Notably, the therapeutic efficiency was comparable to that reported for other colistin-adjuvant combinations (e.g., colistin and auranofin) (25, 41, 42), and considerably higher than those of colistin with cajanin stilbene acid (43) or colistin with a fisetin nanoemulsion (44). As similar therapeutic outcomes in mice have typically required multiple doses of colistin combined with adjuvants administered three times over 3 days (43, 44). These data indicate that the colistin and PFK-158 combination holds considerable promise as a therapeutic strategy to combat MDR pathogens in aquaculture settings.

PFK-158 is an anticancer agent that has completed a phase I clinical trial and has demonstrated safety in mice and mammalian cells (23, 28). At concentrations below 32 μg/ml, PFK-158 had negligible cytotoxicity against RAW 264.7 macrophage cells (28), and its maximum tolerated dose in mice was reported to reach 60 mg/kg (23). In this study, the doses of PFK-158 used were 2–8 μg/ml *in vitro* and 10 mg/kg *in vivo*, which were substantially lower than those of aforementioned studies (23, 28). No obvious side effects were observed in the treated zebrafish (Figure 7). Likewise, colistin doses used here were within the safe ranges established by previous studies (e.g., 10–20 mg colistin/kg *in vivo*) (25, 45, 46). Therefore, these results indicate that the PFK-158 and colistin combination is safe and holds considerable application potential for future use.

Previous studies showed that Gram-negative bacteria develop colistin resistance via multiple mechanisms, including a variety of lipid A modifications (e.g., L-ara4N, phosphoethanolamine) mediated by genes on chromosome (e.g., *mgrB*) or encoded by plasmids (e.g., *mcr-1*), complete LPS depletion, efflux pump activation, formation of capsule, overexpression of some outer membrane proteins, and inactivation of colistin with colistinase (47, 48). Specifically, an increase in the positive charge of lipid A (e.g., L-Ara4N modification) would disrupt the electrostatic interactions between the lipid A moiety and the cationic colistin at the bacterial outer membrane, reducing the bactericidal activity of colistin (48). The *arnBCADTEF* operon and *ugd* are essential genes involved in the modification pathway, and their suppression can increase colistin's affinity for the bacterial membrane and enhance its membrane damaging activity (47–49).

While the molecular mechanism underlying the synergistic action of PFK-158 and colistin remains incompletely elucidated. In the present study, the expression levels of the *arnBCADTEF* operon and *ugd* were significantly downregulated following colistin/PFK-158 treatment (Figure 6D), accompanied by an increase in membrane-bound colistin (Supplementary Figure S5). Similar results were observed in MDR bacteria treated with combinations of 7,8-dihydroxyflavone and colistin, or diethyldithiocarbamate and polymyxin B (30, 50). The *E. piscicida* mutants Δ*ugd* and Δ*arnT* were markedly more sensitive to colistin, with MIC_colistin_ values decreased by 64- and 32-fold, respectively (Supplementary Figure S7). These results suggest that the interaction between colistin and the bacterial membrane is pivotal to the synergistic effect of the colistin/PFK-158 combination, and that the lipid A modification pathway may represent a critical target for re-sensitizing MDR Gram-negative bacteria to colistin. These observations are consistent with previous reports (51, 52), wherein diverse antibacterial agents have been designed to tackle polymyxin resistance by targeting lipid A-modifying pathways (51–53).

We also found that the combined treatment of colistin and PFK-158 induced marked morphological alterations in *E. piscicida* cells (Figure 3G), while the bacterial cell membrane structure remained largely intact across all treated groups (Figure 3H). The observed morphological changes varied from those reported in studies using colistin combinations with auranofin or silver against *E. piscicida* (25, 32), where cells exhibited severely structural damages. Notably, biochemical analysis revealed that the combination significantly enhanced outer membrane permeability (Figure 4), thereby facilitating the intracellular uptake of colistin and the adjuvant into bacterial cells and ultimately increasing antibiotic efficacy Consistent with this, a striking accumulation of intracellular colistin was observed in the colistin/PFK-158 treated groups of PPD 130/91, LY2019 and ZX-1 cells (Figures 5D–F). These findings align with previous reports indicating that colistin combined with berberine and EDTA, or melatonin, enhance OM permeability and intracellular antibiotic accumulation in *Salmonella* without severe impairment of membrane integrity (42, 46). Moreover, the intracellular accumulation of colistin might be may be partially attributed to the inhibitory activity of PFK-158 against bacterial efflux pumps (Figures 5A–C). Interestingly, transcriptomic analysis failed to identify enriched differentially expressed genes (DEGs) associated with efflux pumps (Figure 6). Similar discrepancies have been reported in other investigations (42, 54). The discrepancy between biochemical findings and transcriptomic data may stem from the asynchrony among mRNA transcription levels, protein abundance, and enzyme activity.

**Figure 8:**
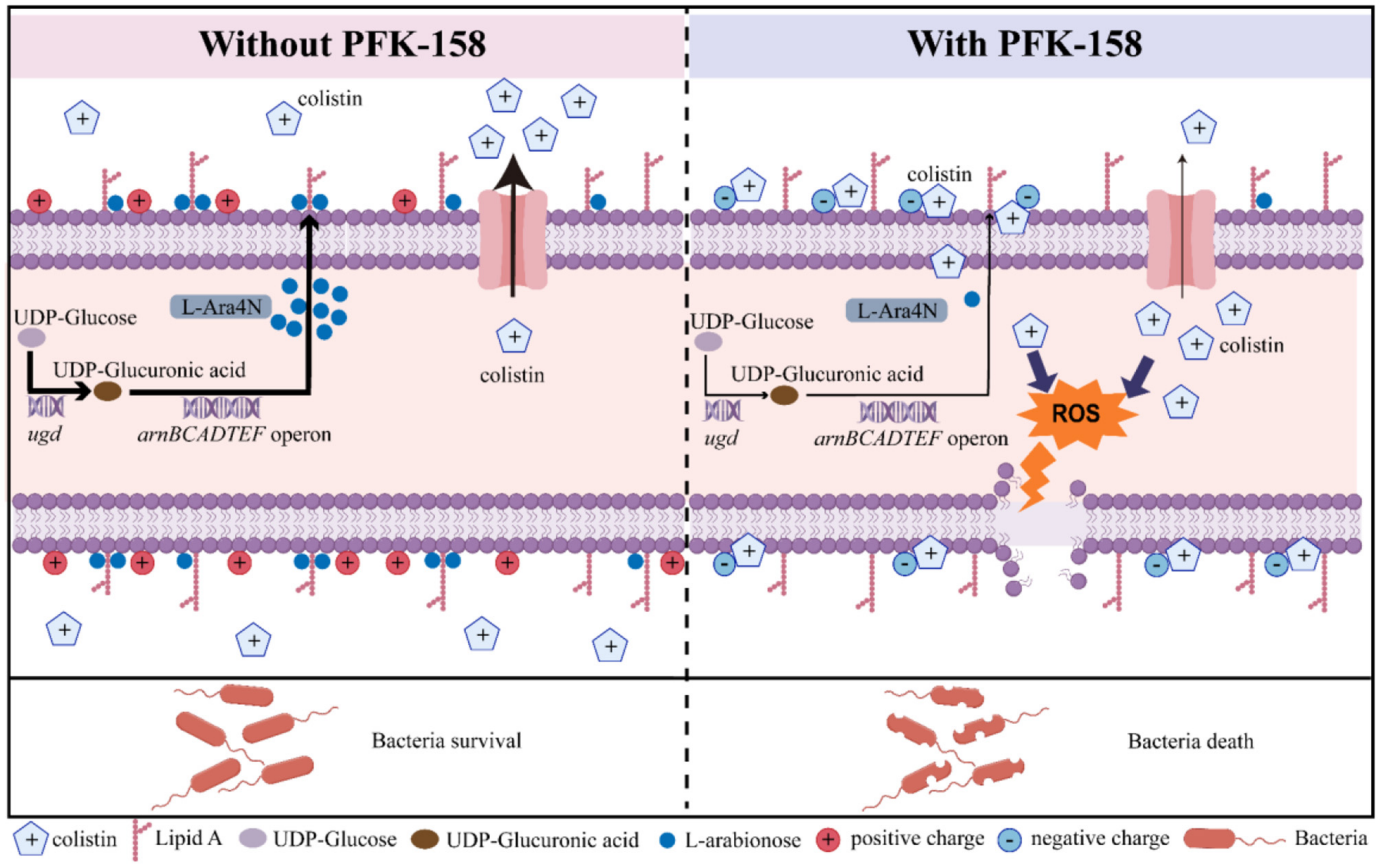
Schematic diagram summarizing the synergistic mechanism of the colistin-PFK-158 combination. The colistin-PFK-158 combination enhances the outer membrane permeability of *E. piscicida*, inhibits bacterial efflux pumps, and downregulates the transcription of genes associated with lipopolysaccharide (LPS) biosynthesis and modification. These effects collectively promote the membrane-binding capacity and intracellular accumulation of colistin, thereby triggering excessive oxidative stress and damage in bacterial cells. Taken together, these synergistic actions significantly augment the antimicrobial efficacy of colistin against colistin-resistant *E. piscicida*.

Elevated intracellular concentration of colistin may promote massive production of ROS (49, 55), thereby triggering extensive oxidative damage. Colistin inhibits the activity of NADH-quinone oxidoreductase (a key respiratory enzyme) (56), and disrupts the chaperone function of heat shock protein 90 (57), thereby exacerbating oxidative stress. Consistent with these previous findings, a significant increase in ROS levels was observed in the combination treatment groups (Figures 5G–I). Supplementation with 5 mM N-acetylcysteine (NAC), a classic antioxidant, remarkably suppressed ROS production (Figures 5G–I), and has been shown to rescue bacterial cells underwent combination treatment (25). These results suggest that oxidative stress plays a vital role in the bactericidal process of colistin/PFK-158 treatment, and that redox cascades could serve as additional potential targets for combining colistin with various adjuvants against MDR Gram-negative bacteria (55, 58). Based on these data, we propose a model illustrating the synergistic mechanism (Figure 8).

Moreover, anti-virulence compounds have been considered as an important reservoir of non-antibiotic adjuvants, and some candidates (e.g., gallium) have been shown to effectively suppress the virulence factors (e.g., biofilm, type III secretion system) and simultaneously reverse bacterial antibiotic resistance (59, 60), thereby improving the efficacy of antibiotics without imposing strong selective pressure for conventional antibiotic resistance. Unexpectedly, the colistin/PFK-158 combination also exhibited both anti-biofilm and anti-virulence effects against *E. piscicida* in this study (Figure 6). Specifically, the combination therapy significantly inhibited the expression of genes related to biofilm formation, T3SS, and T6SS (Figures 6A, B, E). The remarkable therapeutic effects may result from the multi-target activities of the colistin/PFK-158 combination. Notably, the anti-virulence effect of PFK-158 has not been reported previously (23, 28), although the precise molecular targets underlying this activity remain to be elucidated.

The application of antibiotics, particularly colistin, must be strictly regulated in aquaculture due to the potentially serious adverse impacts (e.g., drug residues, neurotoxicity, and facilitation of MDR). Consequently, lots of strict regulatory policies have been established to mitigate these risks (61, 62). Notably, a recent study reported that the reaction-induced self-assembly of polymyxins with natural aldehydes exerts potent bactericidal activity in clinical tests without obvious side effects *in vivo* (63). Moreover, dextrin or mannose-maltose conjugation can effectively reduce colistin toxicity without compromising antimicrobial efficacy (64). Furthermore, numerous adjuvants with multiple targets have been developed (15, 16). Therefore, it is promising to design effective combinations of antibiotics with appropriate adjuvants to address antibiotic resistance in aquaculture.

## 5 Conclusion

In this study, we showed that the potential anticancer drug PFK-158 serves as a robust adjuvant capable of reversing colistin resistance in *E. piscicida* isolates under both *in vitro* and *in vivo* conditions. PFK-158 enhanced the bactericidal activity of colistin by potentiating membrane damage and by inhibiting bacterial efflux pumps as well as biofilm formation. This synergistic effect promotes the intracellular and membrane associated accumulation of colistin, thereby exacerbating oxidative stress in *E. piscicida*. Transcriptomic analysis revealed that the colistin/PFK-158 combination suppressed the processes of lipid A modification, two-component system pathways while concurrently reducing the expression of virulence related genes. Furthermore, PFK-158 combined with colistin effectively reduced the bacterial loads in the liver, spleen, kidney of zebrafish, which in turn ameliorated the survival outcomes. Collectively, these results demonstrate that PFK-158 is a promising antibacterial and anti-virulence adjuvant that acts synergistically with colistin against colistin-resistant Gram-negative bacteria. These findings facilitate the development of alternative therapeutic strategies to combat infections caused by MDR bacteria in both aquaculture and clinical settings.

## Data Availability

The data presented in the study are deposited in the National Center for Biotechnology Information repository, accession number PRJNA1417815.
