# Correction: PFK-158 enhances colistin efficacy against resistant *Edwardsiella piscicida* through synergistic mechanisms

**DOI:** 10.3389/fvets.2026.1817021

**Published:** 2026-03-13

**Authors:** Yajing Pan, Yuepeng Zhang, Zubair Ahmed Laghari, Yangbin Shi, Hans-Peter Grossart, Yelin Jiang, Sihong Wu, Danli Xie, Wanchun Guan, He Zhang, Yongliang Lou, Jinfang Lu

**Affiliations:** 1 Wenzhou Key Laboratory of Sanitary Microbiology, Key Laboratory of Laboratory Medicine, Ministry of Education, China, School of Laboratory Medicine and Life Sciences, Wenzhou Medical University, Wenzhou, Zhejiang, China; 2 Cangnan Ecological Environmental Monitoring Station, Wenzhou Bureau of Ecology and Environment Cangnan Branch, Wenzhou, Zhejiang, China; 3 Department of Veterinary Parasitology, Sindh Agriculture University, Tandojam, Sindh, Pakistan; 4 Leibniz-Institute of Freshwater Ecology and Inland Fisheries (IGB), Stechlin, Germany; 5 Institute of Biochemistry and Biology, University of Potsdam, Potsdam, Germany; 6 Zhejiang Provincial Key Laboratory for Subtropical Water Environment and Marine Biological Resources Protection, National and Local Joint Engineering Research Center of Ecological Treatment Technology for Urban Water Pollution, College of Life and Environmental Sciences, Wenzhou University, Wenzhou, Zhejiang, China

**Keywords:** aquaculture, *Edwardsiella piscicida*, non-antibiotic adjuvant, PFK-158, synergistic effect

Author He Zhang was erroneously assigned to affiliations “^4^Leibniz-Institute of Freshwater Ecology and Inland Fisheries (IGB), Stechlin, Germany and ^5^Institute of Biochemistry and Biology, University of Potsdam, Potsdam, Germany.”

These affiliations have now been removed for author He Zhang.

The correct affiliation is “He Zhang^6*^.”

The original version of this article has been updated.

