# Supplementary material for: PFK-158 enhances colistin efficacy against resistant Edwardsiella piscicida through synergistic mechanisms
Source: Front Vet Sci. 2026 Feb 12;13:1748700. doi: 10.3389/fvets.2026.1748700 (PMC12935630; doi:10.3389/fvets.2026.1748700)
Supplement: Supplementary file 1 [file Data_Sheet_1.doc]

**Supplementary Materials**

**PFK-158 Enhances Colistin Efficacy Against Resistant *Edwardsiella piscicida* Through Synergistic Mechanisms**

Yajing Pan^1#^, Yuepeng Zhang^2#^, Zubair Ahmed Laghari^3^, Yangbin Shi^1^, Hans-Peter Grossart^4^, Yelin Jiang^1^, Sihong Wu^1^, Danli Xie^1^, Wanchun Guan^1^, He Zhang^4*^, Yongliang Lou^1*^ Jinfang Lu^1**^

^1^Wenzhou Key Laboratory of Sanitary Microbiology, Key Laboratory of Laboratory Medicine, Ministry of Education, China, School of Laboratory Medicine and Life Sciences, Wenzhou Medical University, Wenzhou, Zhejiang 325035, China

^2^Wenzhou Cangnan Ecological Environmental Monitoring Station, Wenzhou, Zhejiang 325800, China

^3^ Department of Veterinary Parasitology, Sindh Agriculture University, Tandojam 70060, Sindh, Pakistan

^4^Leibniz-Institute of Freshwater Ecology and Inland Fisheries (IGB), Stechlin 16775, Germany; University of Potsdam, Institute of Biochemistry and Biology, Potsdam 14469, Germany

^5^Zhejiang Provincial Key Laboratory for Subtropical Water Environment and Marine Biological Resources Protection, National and Local Joint Engineering Research Center of Ecological Treatment Technology for Urban Water Pollution, College of Life and Environmental Sciences, Wenzhou University, Wenzhou, Zhejiang 325035, China

* Correspondence: He Zhang, zhanghe@wzu.edu.cn; Yongliang Lou, lyl@wmu.edu.cn;

Jinfang Lu, jflu@wmu.edu.cn

# These authors contributed equally to this work.

**This supplementary information contains:**

⚫ 5 Texts

⚫ 3 Tables

⚫ 7 Figures

**Text list:**

**Text S1** Chemicals and Reagents

**Text S2** Construction of *E. piscicida* mutants

**Text S3** Bacterial biofilm analysis

**Text S4** Colistin binding assay

**Text S5** Transcriptome analysis and qRT-PCR validation

**Table list:**

**Table S1** The MIC values of each candidate adjuvant against three *E. piscicida* isolates.

**Table S2** Strains and plasmids used in this study

**Table S3** Primers used in this study

**Figure list:**

**Figure S1** The combination of PFK-158 and colistin significantly restores the colistin sensitivity against various colistin-resistant strains.

**Figure S2** Synergistic activity of the combination of PFK-158 and colistin against colistin-resistant *E. piscicida* PPD130/91 in host-mimicking medium.

**Figure S3. Effect of PFK-158 and colistin on transcriptional changes in *E. piscicida* and qRT-PCR validation.**

**Figure S4** Colistin combined with PFK-158 suppresses biofilm formation of colistin-resistant *E. piscicida*. (****p* < 0.001).

**Figure S5** PFK-158 enhances colistin binding to the bacterial membrane in a dose-dependent pathway. ppb, parts per billion. Significant differences were evaluated by two-way ANOVA analysis (**p* < 0.05, ****p* < 0.001,).

**Figure S6. Combined treatment with colistin and PFK-158 ameliorated the clinical signs of infected zebrafish.**

**Figure S7** Synergistic activity of the colistin-PFK-158 combination against two *E. piscicida* mutants (*ΔarnT*, *Δugd*), which are deficient in LPS modification-related genes.

**Text S1 Chemicals and reagents**

Tryptic Soy Broth (TSB) and cation-adjusted Mueller-Hinton broth (CAMHB) were obtained from BD Biosciences (San Jose, CA, USA) and Hopebio (Qingdao, China), respectively. Natural metabolites including (-)-Epigallocatechin Gallate sulfate (EGCG), quercetin, myricetin, along with synthetic compounds such as carbonyl cyanide 3-chlorophenylhydrazone (CCCP), Phe-Arg-β-naphthylamide dihydrochloride (PAβN), PFK-158 were purchased from MedChem Express (Shanghai, China). 1-*N*-phenylnaphthylamine (NPN) was obtained from Aladdin Bio-Chem Technology Co. LTD (Shanghai, China). The enzyme-linked immunosorbent assay (ELISA) kit for colistin detection was obtained from Abebio (Wuhan, China). Dulbecco’s Modified Eagle Medium (DMEM) medium and fetal bovine serum (FBS) were purchased from Biological Industries (Northern Kibbutz Beit Haemek, Israel). The Bacteria RNA Extraction Kit, PrimeScript™ RT reagent kit with gDNA eraser, and SYBR Mix kit were purchased from Vazyme (Nanjing, China), Takara (Dalian, China), and Qingke (Beijing, China), respectively. Propidium iodide (PI), Hoechst 33342 (H33342), N-Acetyl-L-cysteine (NAC), 4-(2-Hydroxyethyl)-1-piperazineethanesulfonic acid (HEPES), cell counting kit-8 (CCK-8), trypsin/EDTA, and 2′, 7′-dichlorodihydrofluorescein diacetate (DCFH-DA), all were purchased from Beyotime (Shanghai, China).

**Text S2 Construction of mutants**

The in-frame deletion mutants of *arnT* (ETAE_1295) and *ugd* (ETAE_1202) were constructed using a *sacB*-based allelic exchange method as described by Lu et al. (2025) (1). Briefly, the upstream and downstream flanking regions of each target gene were amplified by polymerase chain reaction (PCR), and the two resulting fragments were fused via a second overlapping PCR. The resulting amplicon, comprising the contiguous flanking regions without the entire coding sequence of the target gene, was inserted into a suicide vector pRE112, and then transferred into *E. coli* S17-1 *λpir* for subsequent conjugation with *E. piscicida* PPD130/91. The mutant strains, Δ*arnT* and Δ*ugd* were successfully constructed and verified by PCR. The primers used in this study are listed in Table S3.

**Text S3 Biofilm formation inhibition assay**

The biofilm inhibition assay was conducted as previously described by Lu et al., (2025) (1). Briefly, mid-logarithmic phase *E. piscicida* cultures were treated with PFK-158, colistin, or the combination of colistin and PFK-158 for 24 hours at 28 °C to allow biofilm formation. Subsequently, the medium and planktonic bacteria were removed, and remaining bacterial cells were rinsed thrice with PBS buffer. The plates were then allowed to air- dry at room temperature for 30 min and stained with 1 % crystal violet (37 °C, 15 min). Finally, 200 μL of destaining solutions (95 % ethanol and 5 % acetic acid) was added into each well and incubated at 37 °C for 30 min to solubilize the stained biofilm. The optical absorbance was measured at 595 nm using a microplate reader, with higher OD₅₉₅ values indicating greater biofilm biomass. All experiments were performed with at least three independent biological replicates.

**Text S4 Colistin binding assay**

The binding affinity of colistin to the bacterial membrane was assessed after treatment with PFK-158 using a method from previous study (2). Briefly, *E. piscicida* PPD130/91 at mid-logarithmic growth phase was incubated with colistin for 30 min at 28°C in the presence of various concentrations of PFK-158 (0, 2, 4, 8, 16 μg/mL). Cells were collected and then washed three times with normal saline solution (0.9%, NaCl) to remove unbound extracellular colistin. To dissociate colistin specifically bound to the bacterial cell membrane, the cell pellets were resuspended in 200 μL of glycine-HCl buffer (pH 2.4) and incubated at 28 °C for 30 min. The resulting supernatants were collected by centrifugation for 5 min at 12, 000 × g at 4 °C, and then 1 M Tris (1:25 for Tris: glycine-HCl) was added. The concentration of dissociated colistin was quantified using a Colistin ELISA Kit (Abebio, Wuhan, China) according to the manufacturer’s instructions.

**Text S5 Transcriptome analysis and qRT-PCR validation**

Total RNA of *E. piscicida* PPD130/91 was extracted using the EASY Spin Plus Bacterial RNA extraction kit (Aidlab, Beijing, China) following the manufacturer’s protocol. RNA purity and quantification were detected using a NanoDrop 2000 spectrophotometer (Thermo Scientific, MA, USA). RNA integrity was evaluated using an Agilent 2100 Bioanalyzer (Agilent Technologies, CA, USA). Ribosomal RNA (rRNA) was depleted using the RiboCop rRNA Depletion Kit for Mixed Bacterial Samples (lexogen, USA). The resulting pure mRNA was fragmented into short fragments (~200 nt), and the RNA-seq transcriptome libraries were constructed using the TruSeq RNA Sample Prep Kit (Illumina Inc., CA, USA) according to the manufacturer’s instructions. Paired-end sequencing of the libraries was performed on an Illumina NovaSeq 6000 platform (Illumina Inc., CA, USA) with a sequencing depth of approximately 1000×. Clean reads were generated by filtering out low-quality reads, adapters, and contaminating sequences, then mapped to the reference genome of *Edwardsiella piscicida* EIB202 using HISAT2 (version 2.0). Gene transcriptional abundance was normalized the Fragments Per Kilobase of transcript per Million mapped reads (FPKM) method. Differentially expressed genes (DEGs) between treatment groups were identified using the DESeq2 package (version 1.36.0). Genes with an absolute fold change (|FC|) ≥ 2 and a Bonferroni-corrected P-value (Padj) < 0.05 were defined as significant DEGs. Finally, significant DEGs were subjected to Gene ontology (GO) and Kyoto Encyclopedia of Genes and Genomes (KEEG) pathway enrichment analyses to explore their biological functions.

Quantitative real-time polymerase chain reaction **(**qRT-PCR) analysis was performed as previously described to validate the transcriptional level of several representative DEGs identified by transcriptome analysis (1). Briefly, *E. piscicida* PPD130/91 at mid-logarithmic growth phase (OD_540_ = 0.5) was diluted 1:10, and treated with colistin (16 μg/mL) alone, or with the colistin/PFK-158 combination for 4 hours, respectively. After that the total RNA was extracted using a Bacterial RNA Extraction Kit (Vazyme, Nanjing, China), and then 1 μg of RNA was reverse-transcribed to cDNA using a PrimeScript™ RT reagent Kit with gDNA Eraser (Takara, Dalian, China). qRT-PCR was performed in a CFX96 Real-Time PCR Detection System (Bio-Rad, CA, USA) using gene-specific primers (Table S3), and SYBR Mix kit (Qingke, Beijing, China). The *16S rRNA* was used as the endogenous reference gene, and the relative expression level of each gene was calculated using the 2^-ΔΔCT^ method (3). Each qRT-PCR assay was performed with three biological replicates and three technical replicates per biological sample. Statistical significance was analyzed by one-way analysis of variance (ANOVA), with *p* < 0.05 considered statistically significant.

**Table S1.** The MIC values of each candidate adjuvant against three *E. piscicida* isolates.

| Species | Strains | MIC values (μg/mL) | | | | | | |
| --- | --- | --- | --- | --- | --- | --- | --- | --- |
|  |  | EGCG | | Que | Myr | CCCP | PAβN | PFK-158 |
| *E. piscicida* | PPD130/91 | | 64 | 128 | 64 | 4 | 256 | 256 |
|  | LY-2019 | 128 | | 256 | 128 | 2 | 256 | 256 |
|  | ZX-1 | 128 | | 256 | 128 | 2 | 256 | 256 |

EGCG, (-)-epigallocatechin gallate sulfate; Que, quercetin; Myr, myricetin; CCCP, carbonyl cyanide 3-chlorophenylhydrazone; PAβN, phe-arg-β-naphthylamide dihydrochloride.

**Table S2**. Strains and plasmids used in this study

| Strains or plasmids | Description | Reference or source |
| --- | --- | --- |
| Strains |  |  |
| *E. piscicida* |  |  |
| PPD130/91 | wild-type, Kan^s^, Col^r^, Amp^s^ | [4] |
| Δ*arnT* | PPD130/91 with in-frame deletion mutation of *arnT*, Col^s^ | This study |
| Δ*ugd* | PPD130/91 with in-frame deletion mutation of *ugd*, Col^s^ | This study |
| LY-2019 | wild-type, Col^r^ | This study |
| ZX-1 | wild-type, Col^r^ | This study |
| *Edwardsiella ictaluri* | wild-type, Col^r^ | [5] |
| *Edwardsiella anguillarum* | wild-type (CCTCC AB 2013118), Col^r^ | [6] |
| *Vibrio parahaemolyticus* |  |  |
| 2017-34 | wild-type, Col^r^ | This study |
| 2016-78 | wild-type, Col^r^ | This study |
| 2016-73 | wild-type, Col^r^ | This study |
| *Vibrio vulnificus* | wild-type, Col^r^ | [7] |
| *Escherichia coli* |  |  |
| DH5α | α complementation | Stratagene |
| S17-1 *pir* | RK2 *tra* regulon, *pir*, host for *pir*-dependent plasmid | [8] |
| Plasmids |  |  |
| pMD18-T | Cloning vector, Amp^r^ | TaKaRa |
| pRE112 | Suicide plasmid, *pir* dependent, Cm^r^, *oriT*, *oriV*, *sacB* | [9] |
| pRE-Δ*arnT* | pRE112 with *arnT* flanking fragments | This study |
| pRE-Δ*ugd* | pRE112 with *ugd* flanking fragments | This study |
| pJN-105 | Arabinose-inducible gene expression vector; *araC*-P_BAD_; Gm^r^ | [10] |
| pJN-*mcr*-1 | pJN-105 with *mcr*-1 | This study |

Col, colistin; Kan, kanamycin; Amp, ampicillin; Tet, tetracycline; Cm, chloramphenicol;

Superscripts: r, resistance; s, sensitivity

**Table S3.** Primers used in this study

| Primers | Primer sequences | Reference | |
| --- | --- | --- | --- |
| *mcr-1*-F | 5'-TGCGCCGATTGGGCTTGATCGTGGC -3' | | [11] |
| *mcr-1*-R | 5'-ATCATAGGCATTGCTGTGCGTCTGC-3' | |
| *mcr*-*2*-F | 5'-GCGATGGCGGTCTATCCTGTATCGG-3' | |
| *mcr*-*2*-R | 5'-GGCTGACACCCCATGTCATCGCACG-3' | |
| *mcr*-*3*-F | 5'-TTGCACTTCTTATCGCACTTAGTGC-3' | |
| *mcr*-*3*-R | 5'-TGGGCTTACTTTGATTAGTATCCCG-3' | |
| *mcr*-*4*-F | 5'-TCTCAGTGAAGTGGTGAATAAATTAAAA-3' | |
| *mcr*-*4*-R | 5'-GCAACACAAGTCATGTTCAACTGATTA-3' | |
| *mcr*-*5*-F | 5'-CTTGAAAATGCGCCCGCAAGTGCGC-3' | |
| *mcr*-*5*-R | 5'-CGGCGGCCTTGTTCTTGAGGCCCTC-3' | |
| *mcr-6-F* | 5'-GTCCGGTCAATCCCTATCTGT-3' | | [12] |
| *mcr-6-R* | 5'-ATCACGGGATTGACATAGCTAC-3' | |
| *mcr*-*7*-F | 5'-ATGCGCATCACGCTCGGTGTGATGAA-3' | | [13] |
| *mcr*-*7*-R | 5'-CTGCACGGTGCGGCACTGGCTGAAAA-3' | |
| *mcr*-*8*-F | 5'-ATGTTCAAGTATCTTTTATCTTTCAA-3' | |
| *mcr-8-R* | 5'-ACCATTCCCATCTGTTTTCTCTCTTA-3' | |
| *mcr-9-F* | 5'-TCAGGGTGAAAGTTATTCCG-3' | |  |
| *mcr-9-R* | 5'-CCAGCGTCTATAATCCTGAC-3' | |
| *mcr-10-F* | 5'-GGGTAATCCCCTTGGTTTTA-3' | |
| *mcr-10-R* | 5'-CAGGACATATTCCCACGATA-3' | |
| mut-*arnT*-F | 5'-GGTACCCTTAAGCCGCGCTTTCTGTGGAAAA-3' | | This study |
| mut-*arnT*-i-R | 5'-ATCAGCAGATAACTCATTCATGGCGACTCCACCTCCTGCTGA-3' | |
| mut-*arnT*-i-F | 5'-ATGAGTTATCTGCTGATTATTTTCT-3' | |
| mut-*arnT*-R | 5'-GGTACCCCAGTAAACCCAGTATAATCAAGCC-3' | |
| mut-*arnT*-c-F | 5'-GGTTTACACCATTCACGCCGAAGTT-3' | |
| mut-*arnT*-c-R | 5'-TAAGCACCACCAGCCACAGCAGTAG-3' | |
| mut-*ugd*-F | 5'-GGTACCGCTTTATTTTTAGCTCTTCTGCGAC-3' | |
| mut-*ugd*-i-R | 5'-AAATTTGCCGGTAATGGCACATGCCTTTGTGTTAATAAAATT-3' | |
| mut-*ugd*-i-F | 5'-CCATTACCGGCAAATTTTGTAAGAC-3' | |
| mut-*ugd*-R | 5'-GGTACCCGGCAGTCCGTACAGATGAGAGTAG-3' | |
| mut-*ugd*-c-F | 5'-TTGAGAGGTGCTCTGGGGTTACTAT-3' | |
| mut-*ugd*-c-R | 5'-ATCGTAATAGTCGTTCAGGTTGTCG-3' | |
| 16s-qPCR-F | 5'-ACTGAGACACGGTCCAGACTCCTAC-3' | |
| 16s-qPCR-R | 5'-TTAACGTTCACACCTTCCTCCCTAC-3' | |
| *ugd*-qPCR-F | 5'-GCTCAACAGCCGTCAGATTATTG-3' | |
| *ugd*-qPCR-R | 5'-AAGTTATCCGAACCGCTCTTCA-3' | |
| *arnT*-qPCR-F | 5'-GTATTACCTGCCGATCCTCCTTC-3' | |
| *arnT*-qPCR-R | 5'-CTGAACAGCAGAAATGGCATCAC-3' | |
| *arnB*-qPCR-F | 5'-TCAGGCGTTGGAGCAAGAGT-3' | |
| *arnB*-qPCR-R | 5'-GGCGTCACCATCAGCGTATC-3' | |
| *arnC*-qPCR-F | 5'-ACCGCAGCAGCATCATCAAG-3' | |
| *arnC*-qPCR-R | 5'-AGGTCACCAGGTCGTACATCAG-3' | |
| *arnA*-qPCR-F | 5'-CTGCTGGCGGAAGATAACTATGAA-3' | |
| *arnA*-qPCR-R | 5'-CAGAGGTGGACGGGAAGATGA-3' | |
| *arnD*-qPCR-F | 5'-ACCGCTTCATTCTCGACAGTATC-3' | |
| *arnD*-qPCR-R | 5'-TCCACCTCCTGCTGACAACC-3' | |
| *arnE*-qPCR-F | 5'-CGCAGGACAGCTATGTCAGAA-3' | |
| *arnE*-qPCR-R | 5'-AGTTCAGGCTCAGCATCGGA-3' | |
| *arnF*-qPCR-F | 5'-GGCGGCATTCTGTGCTATGG-3' | |
| *arnF*-qPCR-R | 5'-CTAAGGCTCAGCAATGGATAGGC-3' | |
| *eseB*-qPCR-F | 5'-CCAACGGCTACGACAACAAC-3' | |
| *eseB*-qPCR-R | 5'-ATTAGCCACCTGCTGGGAGT-3' | |
| *eseC*-qPCR-F | 5'-ATAGACTGGTACTTTTCCGC-3' | |
| *eseC*-qPCR-R | 5'-TTGGCAAAATCTTAATCGGC-3' | |
| *eseD*-qPCR-F | 5'-AAAGTCTTTATCGATGGCGT-3' | |
| *eseD*-qPCR-R | 5'-GATGGTTCAGCTTGGAGAG-3' | |
| *evpB*-qPCR-F | 5'-ATGACAGTGACTCCGCCAAT-3' | |
| *evpB*-qPCR-R | 5'-CATCCGAGCCGACCACATA-3' | |
| *evpC*-qPCR-F | 5'-TACAAATTTACGCATGTCGC-3' | |
| *evpC*-qPCR-R | 5'-GATCCTGGGGAACGTATTC-3' | |
| *evpP*-qPCR-F | 5'-AGTCCTAAAGATTCCCGTCT-3' | |
| *evpP*-qPCR-R | 5'-AATATAGAACTGTGTGGCCC-3' | |
| *ompR*-qPCR-F | 5'-AAACTCGCCGCTGGTCAA-3' | |
| *ompR*-qPCR-R | 5'-AGAGGACGCCGTGATTAACTT-3' | |
| *3304*-qPCR-F | 5'-TATCTGGTGCTGGCTGACTTC-3' | |
| *3304*-qPCR-R | 5'-CCGTGTTGAGGATGGTCTTCT-3' | |
| *appB*-qPCR-F | 5'-GCGGCGAATGGATGAAGTTAC-3' | |
| *appB*-qPCR-R | 5'-TGGTGGTGCTCTGCTTCCT-3' | |
| *bcsA*-qPCR-F | 5'-ACAGGAGCCACAGAAGAAGG-3' | |
| *bcsA*-qPCR-R | 5'-TTACAAACGCCGCACCATTT-3' | |
| *esrA*-qPCR-F | 5'-AACAAGAGCGAGCACCTGAA-3' | |
| *esrA*-qPCR-R | 5'-GCGTATTCTCACCGTTCTCCA-3' | |
| *flgB*-qPCR-F | 5'-CGTCGCTGGATCTGCTGTT-3' | |
| *flgB*-qPCR-R | 5'-CGGTGAGGTCGGTCTGATAAC-3' | |
| *fliN*-qPCR-F | 5'-GCCGCACCAAGATGACCAT-3' | |
| *fliN*-qPCR-R | 5'-TCGTTGACCACCACCACCT-3' | |
| *narG*-qPCR-F | 5'-ACTGGCTGGTGTGATTGTAGAA-3' | |
| *narG*-qPCR-R | 5'-CGCACTATGTCGGTCAGGAA-3' | |
| *rpoS*-qPCR-F | 5'-GAGGTGATCCGCTCGTTAAGG-3' | |
| *rpoS*-qPCR-R | 5'-CCATTCGCCTGCCTATCCATAT-3' | |
| *torA*-qPCR-F | 5'-CGGTAGTCGCTGCTGTTGT-3' | |
| *torA*-qPCR-R | 5'-CAGTGGTCGTGGATGATCGT-3' | |


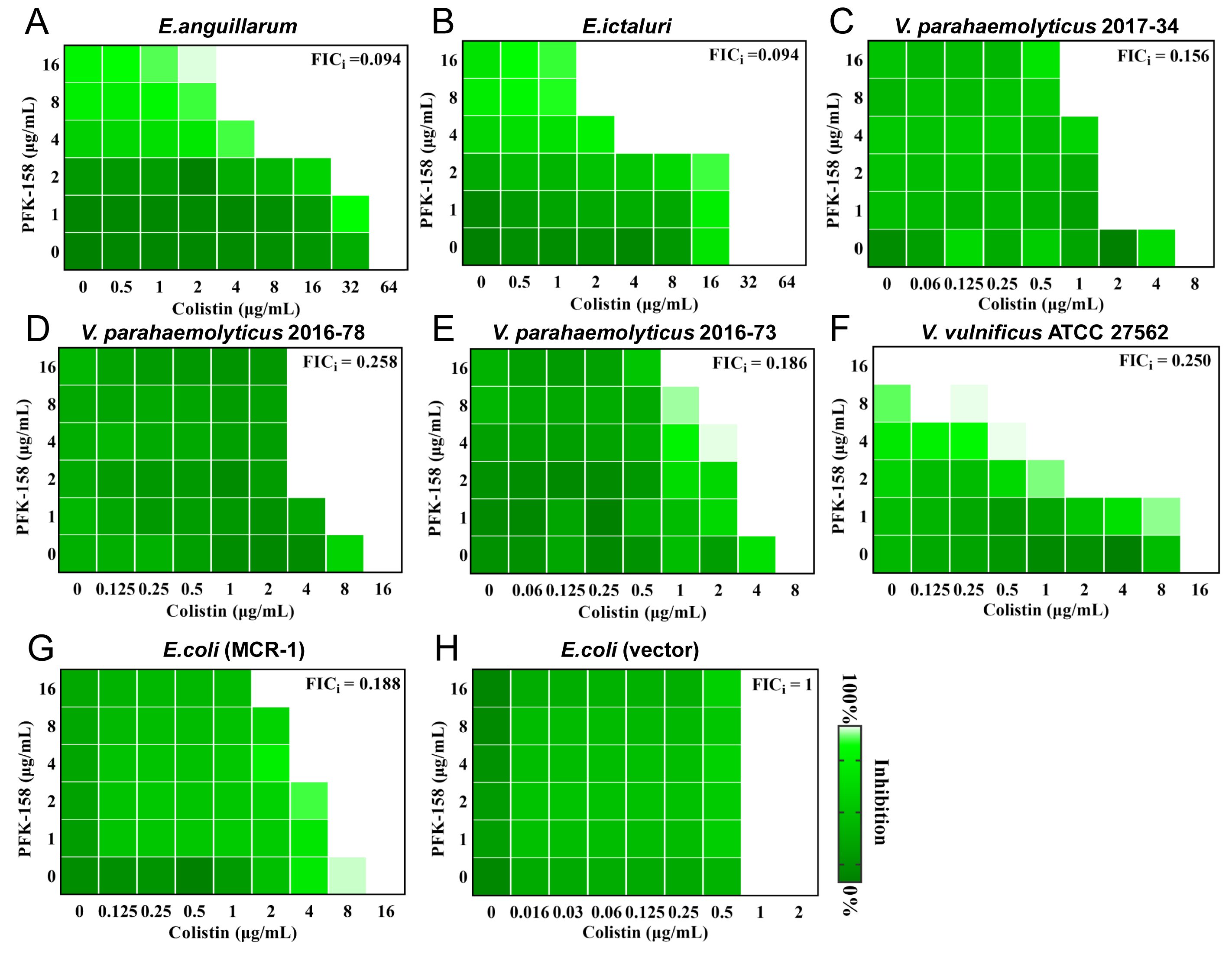


**Figure S1. The colistin/PFK-158 combination significantly restores colistin susceptibility against various colistin-resistant strains. (A-H)** Heatmaps from micro-dilution checkerboard assays illustrating the interaction between colistin and PFK-158 against different colistin-resistant strains and controls: *E. anguillarum*, *E. ictaluri, V*. *parahaemolyticu*s 2017-34, 2016-78, 2016-73, *V*. *vulnificus* ATCC 27562, *mcr-1* positive strain *E. coli* (MCR-1), and the control strain *E. coli* (vector), which was transformed with the empty expression vector pJN-105.. Dark green regions indicate higher bacterial cell density, corresponding to weaker antibacterial activity. FICi < 0.5 indicates synergy. All assays were performed with three independent biological replicates.


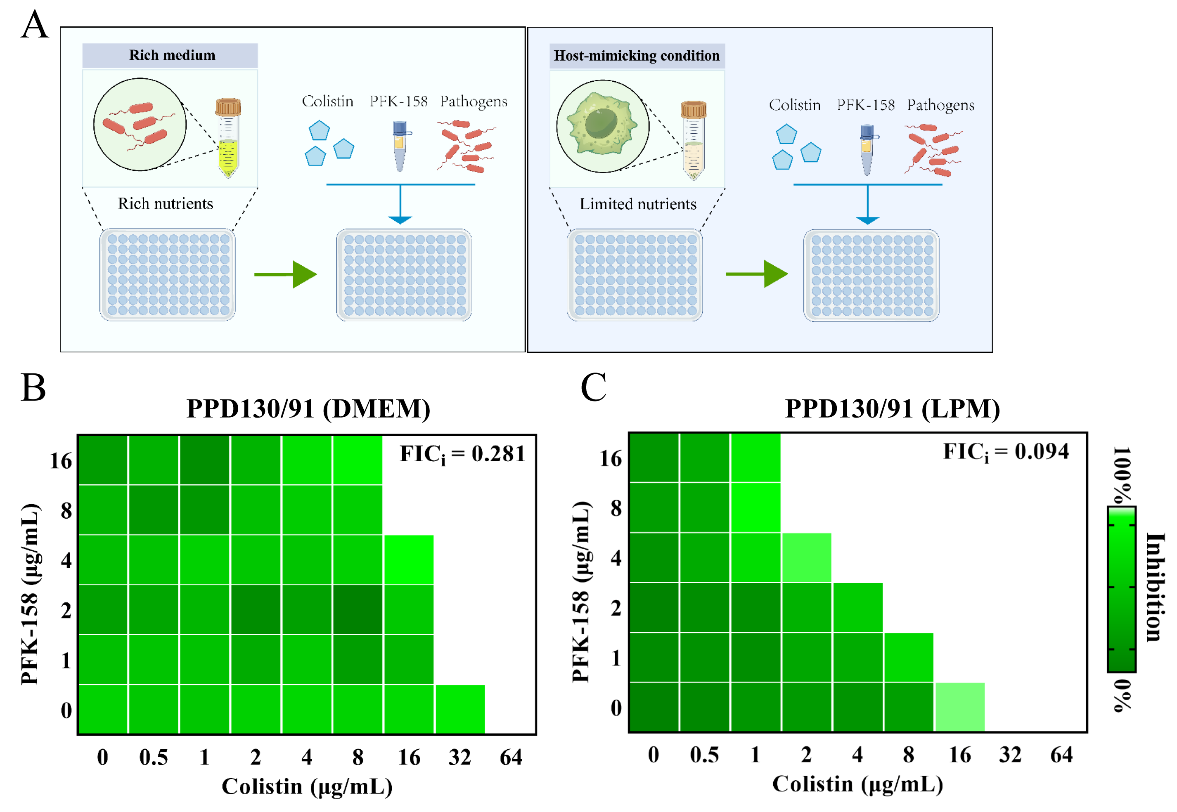


**Figure S2. Synergistic activity of the colistin-PFK-158 combination against colistin-resistant *E. piscicida* PPD130/91 in host-mimicking media.** **(A)** Schematic diagram of the experimental protocol for the checkerboard assay. **(B-C)** Heatmaps from microdilution checkerboard assays showing the interaction between colistin and PFK-158 in Dulbecco’s Modified Eagle Medium (DMEM) and LPM media, respectively. Dark green regions indicate higher bacterial cell density, corresponding to weaker antibacterial activity. FICi < 0.5 indicates synergy. All assays were performed with three independent biological replicates.

**Figure S3. Effect of PFK-158 and colistin on transcriptional changes in *E. piscicida* and qRT-PCR validation. (A)** Top 20 enriched KEGG pathways in *E. piscicida* PPD130/91 exposed to PFK-158 and colistin when compared to colistin alone. **(B)** Selected DEGs involved in two-component system (TCS). **(C)** Relative expression levels of selected DEGs validated via qRT-PCR. Red represents upregulated genes; blue indicates downregulated genes. COL, colistin monotherapy; COL+PFK-158, colistin-PFK-158 combination therapy. Significant differences were evaluated by one-way ANOVA analysis (**p* < 0.05, ***p* < 0.01, ****p* < 0.001, ns means no statistical significance).


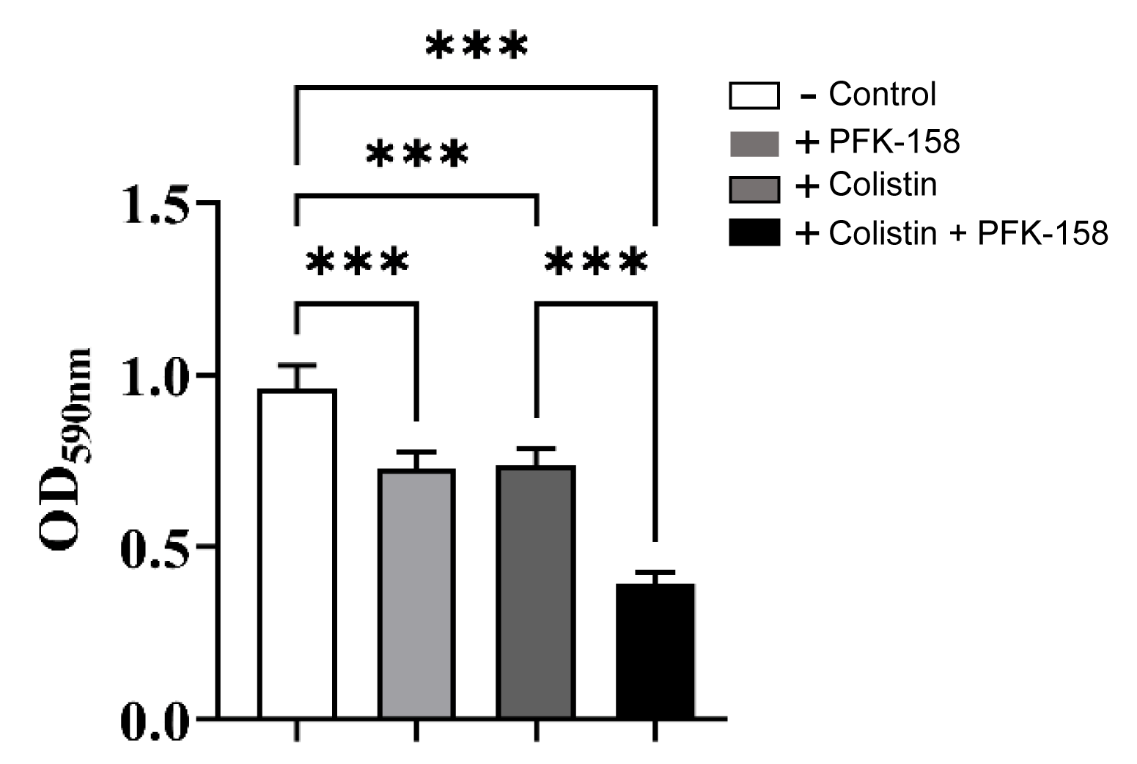


**Figure S4.** The colistin-PFK-158 combination suppresses biofilm formation of colistin-resistant *E. piscicida*. Biofilm biomass was quantified by crystal violet staining, with higher optical density (OD₅₉₅) indicating greater biofilm formation. Significant differences were evaluated by two-way ANOVA analysis (****p* < 0.001, ns means no statistical significance). All assays were performed with three independent biological replicates.


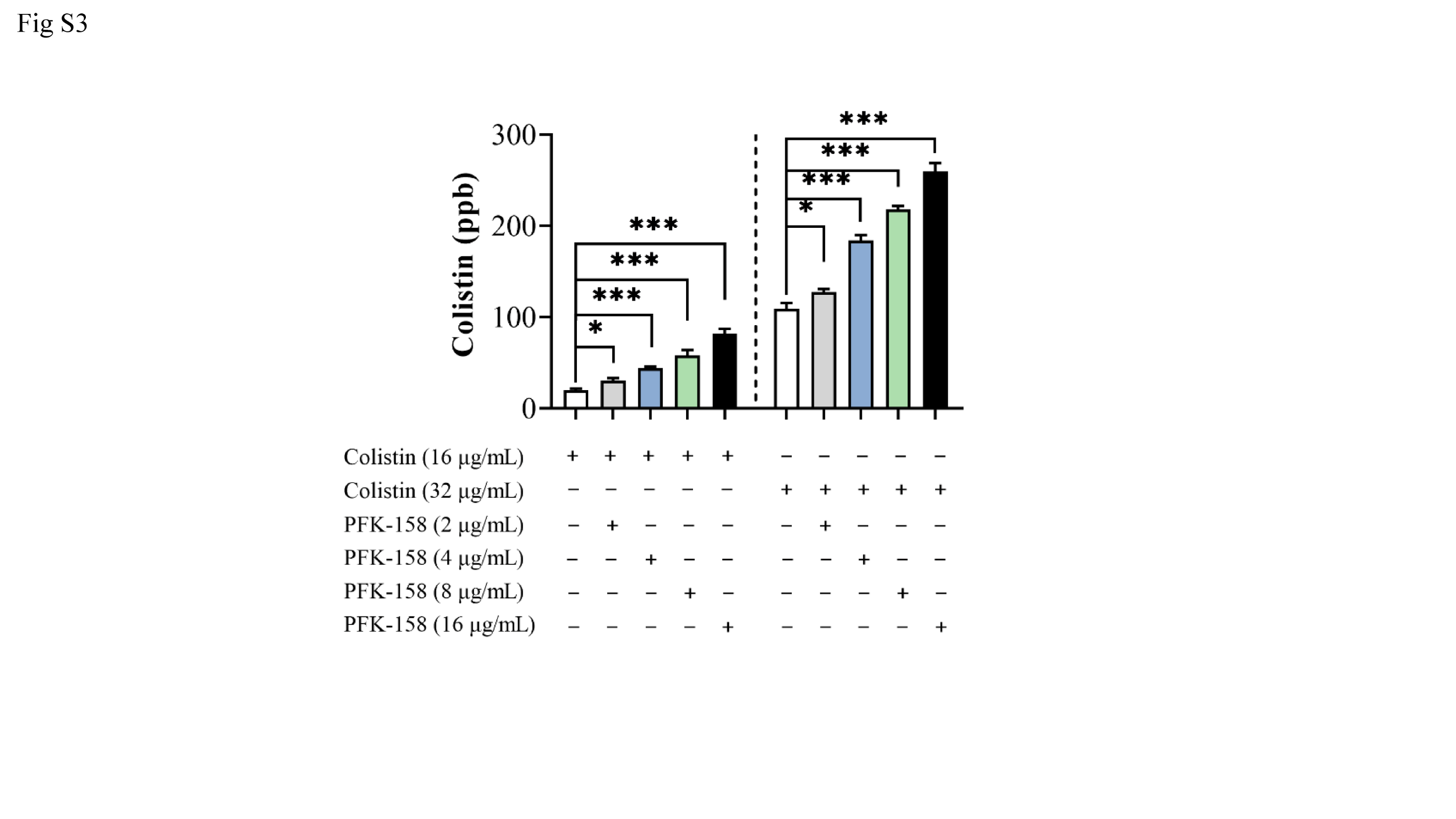


**Figure S5. PFK-158 enhances colistin binding to the bacterial membrane in a dose-dependent manner.** Colistin bound to the bacterial membrane was dissociated and quantified by ELISA, with values expressed in parts per billion (ppb). Higher ppb values indicate increased colistin binding to the membrane. Significant differences were evaluated by two-way ANOVA analysis (**p* < 0.05, ****p* < 0.001). All assays were performed with three independent biological replicates.


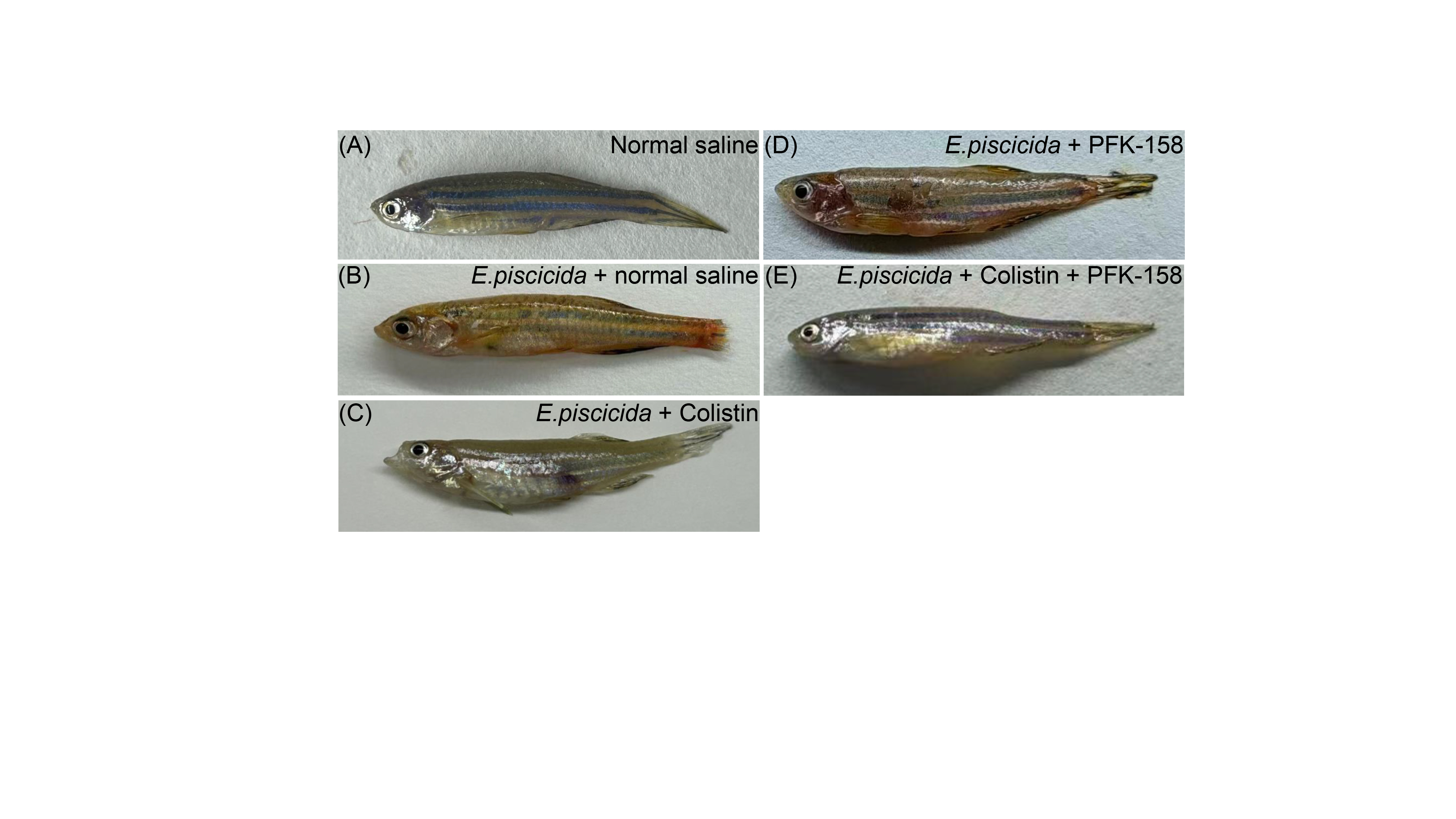


**Figure S6. Combined treatment with colistin and PFK-158 ameliorated the clinical signs of infected zebrafish. (A)** Negative control group; **(B)** Vehicle group; **(C)** Colistin monotherapy group; **(D)** PFK-158 monotherapy group; **(E)** Combinatorial therapy of PFK-158 with colistin group.

**Figure S7.** **Synergistic activity of the colistin-PFK-158 combination against two *E. piscicida* LPS modification-deficient mutants (*ΔarnT*, and *Δugd*). (A–B)** Heatmaps from microdilution checkerboard assays illustrating the interaction between colistin and PFK-158 against *E. piscicida ΔarnT* (A) and *Δugd* (B) mutants, respectively. Deletion of *ugd* or *arnT* directly abrogated the synergistic effect of the colistin-PFK-158 combination (FICi > 0.5). Dark green regions indicate higher bacterial cell density, corresponding to weaker antibacterial activity. FICi < 0.5 indicates synergy. All assays were performed with three independent biological replicates.

**References**

1. Lu J, Shi Y, Pan Y, Hans-Peter Grossart, Mei L, Xie D, et al. Repurposing auranofin combined with colistin to effectively combat fish-pathogenic *Edwardsiella piscicida*. *Aquacult Rep*. (2025) 42:102830. doi:10.1016/j.aqrep.2025.102830.

2. Zhong Z, Zhou S, Liang Y, Wei Y, Li Y, Long T, et al. Natural flavonoids disrupt bacterial iron homeostasis to potentiate colistin efficacy. *Sci Adv*. (2023) 9:4205. doi:10.1126/sciadv.adg4205.

3. Livak KJ, Schmittgen TD. Analysis of relative gene expression data using real-time quantitative PCR and the 2^(-DeltaDelta(CT))^ method. *Methods*. (2001) 25:402–408. doi:10.1006/meth.2001.1262.

4. Lu J, Wang W, Wang G, Zhang H, Zhou Y, Gao Z, et al. *Edwardsiella tarda* EscE (Orf13 protein) is a type III secretion system-secreted protein that is required for the injection of effectors, secretion of translocators, and pathogenesis in fish. *Infect Immun* (2015) 84:2-10. doi:10.1128/IAI.00986-15.

5. Zhao L, Lu J, Nie P, Li A, Xiong B, Xie H. Roles of plasmid-encoded proteins, EseH, EseI and EscD in invasion, replication and virulence of *Edwardsiella ictaluri*. *Vet Microbiol*. (2013) 166:233-241. doi:10.1016/j.vetmic.2013.05.023.

6. Shao S, Lai Q, Liu Q, Wu H, Xiao J, Shao Z, et al. Phylogenomics characterization of a highly virulent *Edwardsiella* strain ET080813 (T) encoding two distinct T3SS and three T6SS gene clusters: propose a novel species as *Edwardsiella anguillarum* sp. nov. *Syst Appl Microbiol*. (2015) 38:36-47. doi:10.1016/j.syapm.2014.10.008.

7. Huang X, Ma Y, Zheng M, Chen N, Hu M, Wu L, et al. NLRP3 and mTOR reciprocally regulate macrophage phagolysosome formation and acidification against *Vibrio vulnificus* infection. *Front Cell Dev Biol*. (2020) 8:587961. doi:10.3389/fcell.2020.587961.

8. Simon R, Priefer U, Pühler A. A broad-host range mobilization system for *in vivo* genetic engineering: transposon mutagenesis in gram negative bacteria. *Biotechnology* (1983) 1:784-791. [doi:10.1038/nbt1183-784](http://dx.doi.org/10.1038/nbt1183-784).

9. Edwards R, Keller L, Schifferli D. Improved allelic exchange vectors and their use to analyze 987P fimbria gene expression. *Gene*. (1998) 207:149-157*.* doi:10.1016/S0378-1119(97)00619-7.

10. Khlebnikov A, Skaug T, Keasling JD*.* Modulation of gene expression from the arabinose-inducible *araBAD* promoter. *J Ind Microbiol Biotechnol.* (2002) 29:34-37. doi:10.1038/sj.jim.7000259.

11. Lei S, Lv J, Gao S, Srinivas S, Feng Y. Developing an efficient multiplex PCR method to detect *mcr*-like genes. *Sci China Life Sci.* (2019) 62:705-707. [doi:10.1007/s11427-019-9512-3](https://doi.org/10.1007/s11427-019-9512-3).

12. Wang X, Wang Y, Zhou Y, Li J, Yin W, Wang S, et al. Emergence of a novel mobile colistin resistance gene, *mcr*-8, in NDM-producing *Klebsiella pneumoniae*. *Emerg Microbes Infect*. (2018) 7:122. [doi:10.1038/s41426-018-0124-z](https://doi.org/10.1038/s41426-018-0124-z).

13. Liao W, Cui Y, Quan J, Zhao D, Han X, Shi Q, et al. High prevalence of colistin resistance and *mcr*-9/10 genes in *Enterobacter* spp. in a tertiary hospital over a decade. *Int J Antimicrob Agents*. (2022) 59:106573. [doi:10.1016/j.ijantimicag.2022.106573](https://doi.org/10.1016/j.ijantimicag.2022.106573).
